# Supplementary material for: C/EBPβ-induced lymphoid-to-myeloid transdifferentiation emulates granulocyte-monocyte progenitor biology
Source: Stem Cell Reports. 2023 Dec 28;19(1):112–25. doi: 10.1016/j.stemcr.2023.11.011 (PMC10828814; doi:10.1016/j.stemcr.2023.11.011)
Supplement: Document S1. Figures S1–S3 and supplemental experimental procedures [file mmc1.pdf]

Stem Cell Reports, Volume *19*

Supplemental Information

C/EBPβ-induced lymphoid-to-myeloid transdifferentiation emulates granulocyte-monocyte progenitor biology

Linh Thuy Nguyen, Karin Zimmermann, Elisabeth Kowenz-Leutz, Ramonique Lim, Maria Hofstätter, Alexander Mildner, and Achim Leutz

Legends to Supplementary Figures, Nguyen et al.

Supplementary Figure S1 (related to Figure 1)

A. Flow cytometric analysis of *Cebpa*$^{fl/fl}$ *Cebpb*$^{fl/fl}$ B cells (WT-B cells) or *Cebpa*$^{-/-}$ *Cebpb*$^{-/-}$ cells (dKO-B cells) transdifferentiation at day 6 p.i. Cells were stained with lineage markers CD19 (B cells), CD11b (myeloid cells), Ly6G (granulocytes) and CD115 (monocytes/macrophages). For scRNA-seq, GFP^{+} populations were sorted as indicated.
B. Expression of top5 differentially expressed marker genes in GMPBT cells. Common genes of Clusters 7 and 8 (*Vpreb1/2/3*, *Ebf1* and *Cd79a*) indicate them as lagging and myeloid primed B cells, respectively.
C. UMAP and clustering of GMPBT derived from dKO- (left, N=1432 cells) and WT-B cells (right, N=3297 cells). All clusters are represented in both samples.
D. Proportion of cells in each cluster comparing WT- and dKO-GMPBT cells. ScRNA-seq data from both WT- and dKO-GMPBT cell pools were integrated and the percentages of cells in each cluster are shown.
E. Expression of the endogenous C/EBP family genes (indicated on top, data derived from scRNA-seq) in myeloid clusters 1-6 are shown for WT- and dKO-GMPBT cells. ****P < 0.0001, ***P < 0.001, **P < 0.01, *P < 0.05, insignificance is not indicated.

Supplementary Figure S2 (related to Figure 2)

A. Gene Set Variation Analysis (GSVA) was applied to compare GMPBT profiling data to published signatures of mouse bone marrow myeloid cells as defined by Giladi et al. (Giladi *et al.*, 2018). Neutrophil signature was used to compute GSVA scores for cells in the GMPBT scRNA-seq data set. Cells with highest scores are indicated in red.
B. Similar to A, monocyte signature from Giladi et al. was used for GSVA.
C. Expression of *Vcam1*, marking pro-neutrophils, across the myeloid Clusters 1-6.

D-G. Gene expression analysis of neutrophil Clusters 1, 2, 3. Genes characteristic for neutrophil progenitors were extracted from Evrard et al. (Evrard *et al.*, 2018), including myeloid transcription factors (D), genes involved in granule production (E), chemotaxis (F, from GO:1903409) and phagocytosis (G, from GO: 0006909).

H. Monocytic gene expression pattern of Clusters 4, 5, 6. Genes identifying monocytes were derived from Mildner et al. (Mildner et al., 2017).

I. GSVA using the signature of DC-like monocytes or Neu-like monocytes, as identified in Weinreb et al. (Weinreb *et al.*, 2020), on monocyte Clusters 4-6. Note that similarities between DC-like monocytes were enriched in Cluster 6, while the Neu-like monocyte signature could be detected preferentially in Cluster 5.

J. Surface marker analysis of GMPBT cells. GMPBT cells transdifferentiated from WT-B cells were subjected to the cell surface marker screening (LegendScreen^{TM}, BioLegend) and processed as described in Materials and Methods. Expressing markers in two independent experiments overlapped, as shown in the Venn's diagram (left). Heatmap (right) shows results of method-optimized experiment 2. The results are presented as percentage of marker-positive cells in each of the GMPBT subsets: Ly6G^{+}, CD115^{+} and double negative (DN).

K. Heatmap representing scRNA-seq expression of genes shortlisted in the heatmap shown in Supplementary Figure S2J. Genes expressed highly in cluster 1 are indicated between the red lines, including *Vcam1*.

L. CD106 (encoded by *Vcam1*) expression in GMPBT subsets as identified in the LegendScreen experiments.

M. Growth curves of isolated CD106 subsets. Two sub-populations of GMPBT cells were sorted: Ly6G^{-}CD115^{-}CD106^{+} (labeled as CD106^{+}) and Ly6G^{-}CD115^{-}CD106^{-} (labeled as CD106^{-}). Sorted cells were seeded at 10^5 cells/mL and cell numbers (N=3) were determined at indicated time points.

N. Differentiation of CD106 subsets. Sorted cells, as shown in Supplementary Figure S2M, were cultivated for 4 days and subjected to flow cytometry analysis for Ly6G and CD115 expression. N=3, P values of Multiple Mann-Whitney t-tests: Ly6G P=0.0286, CD115 P=0.1143.

Supplementary Figure S3 (related to Figure 4)

A. Expression of *Irf8* in scRNA-seq data of WT-GMPBT cells (as in Figure 1A). Note that *Irf8* is expressed in Cluster 4 that relates to the transition/bifurcation of neutrophils and monocyte/macrophages.

B. Representative *Irf8* genotype of Cre-incubated v-Abl transformed *Irf8*$^{fl/fl}$ B cells (PCR analysis). Untreated (-) or Cre-treated cell pool (+Cre) and genotyping of C57BL/6J wild-type mouse (WT) served as controls. Bi-allelic *Irf8*-/- clones (clone 1,2) were subjected to transdifferentiation together with corresponding isogenic control (untreated) clones.

C. Immunoblot analysis of C/EBPβ expression in total protein lysates from C/EBPα,β dKO-B cells transdifferentiated with HA-tagged LAP*-FKBP12^{F36V} (CEBP-dKO-LAP*-FKBP12^{F36V}-GMPBT cells). Cells were treated with 5 µM AP1867, 5 µM FK506, 0.01 µM rapamycin or 5 µM dTAG-13 for 6 hours or 24 hours. Mono-functional AP1867 and FK506 bind to and stabilize FKBP12 while the hetero-bifunctional dTAG-13 selectively degrades the LAP*-FKBP12^{F36V} chimera. Expression of the fusion protein LAP*-FKBP12^{F36V} was detected by an antibody directed against the HA-tag (approx. 55 kDa). Long exposure (top) to demonstrate removal of LAP*-FKBP12^{F36V}. Short exposure (underneath) was used for quantification of LAP*-FKBP12^{F36V} (arbitrary units) after normalization to Poncaeu S-stained lanes (total protein loading/blotting controls; total protein loaded: 25 µg B cells, lane 1, and 100 µg for all other lanes 2-10).

D. Morphology of CEBP-dKO-LAP*-FKBP12^{F36V}-GMPBT cells with or without dTAG-13 treatment. Phase contrast (cell culture samples at day 5 post-treatment, top row) and May-Grunwald Giemsa staining (cytospins of samples at day 1 post-treatment, bottom row) images are shown. Scale bar: 50 µm. Note larger cell size and macrophage appearance after dTAG-13 treatment of GMPBT cells.

Supplemental Figure 1

Supplementary Figure S1

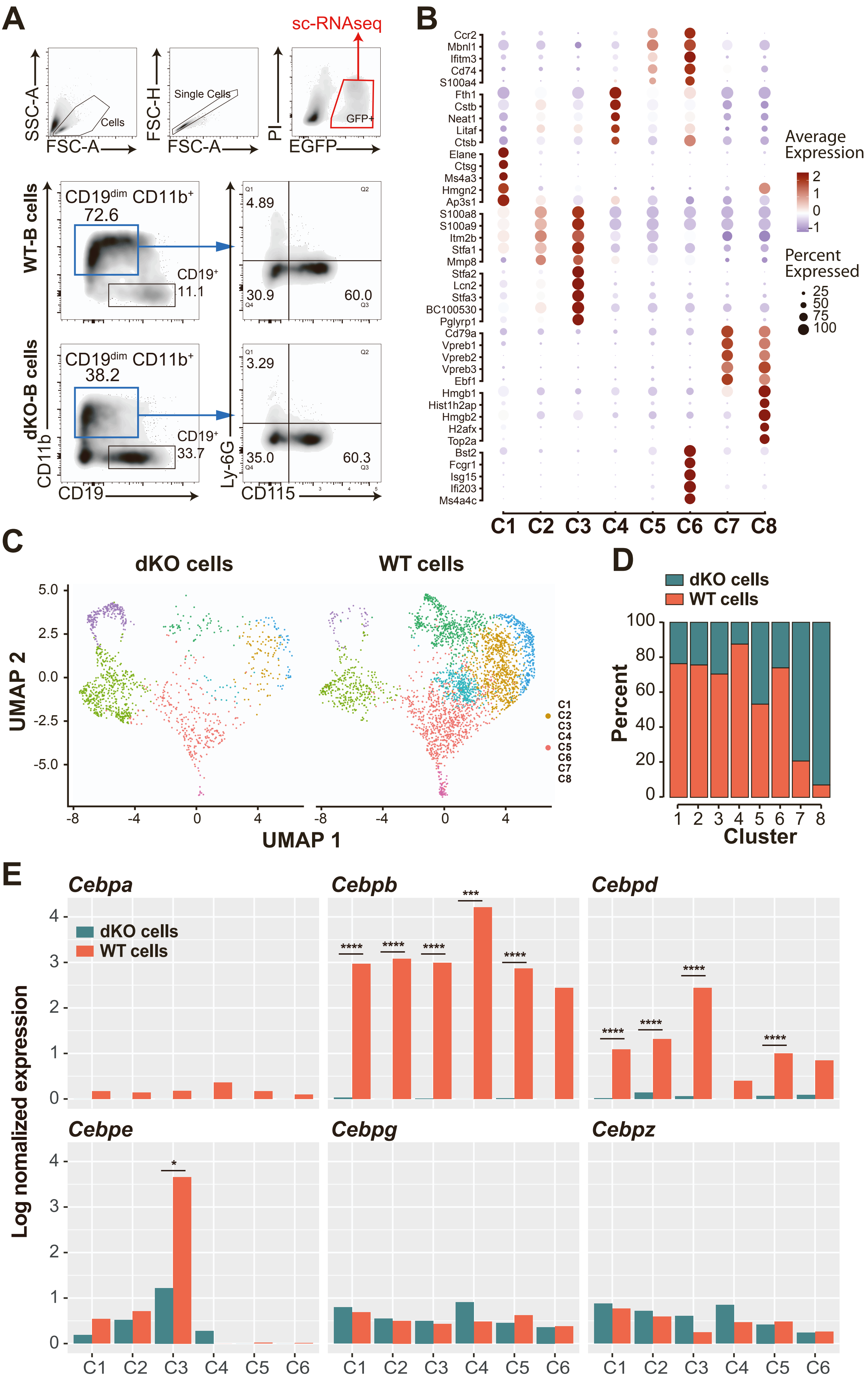

Supplemental Figure 2

Supplementary Figure S2

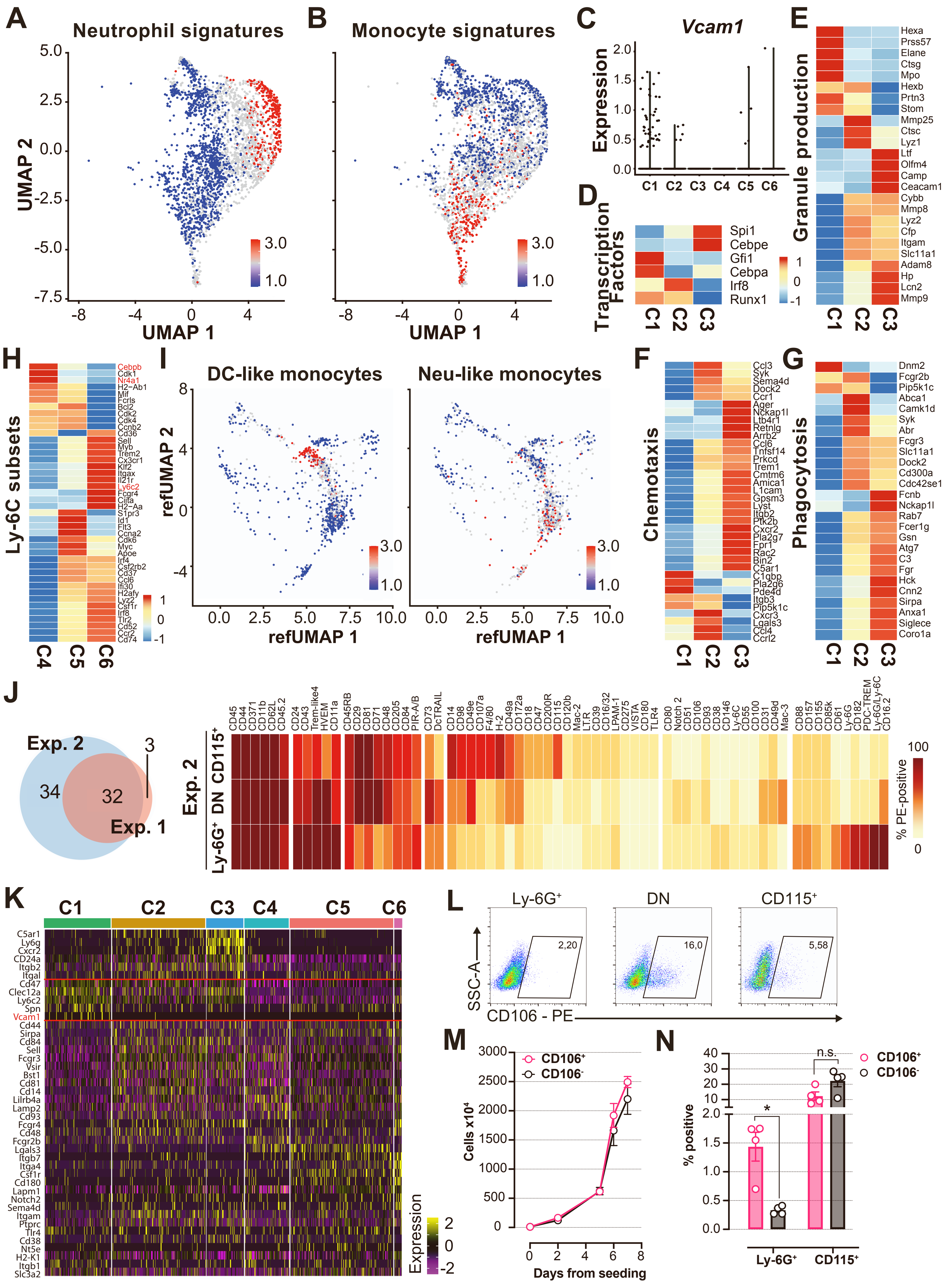

Supplemental Figure 3

Supplementary Figure S3

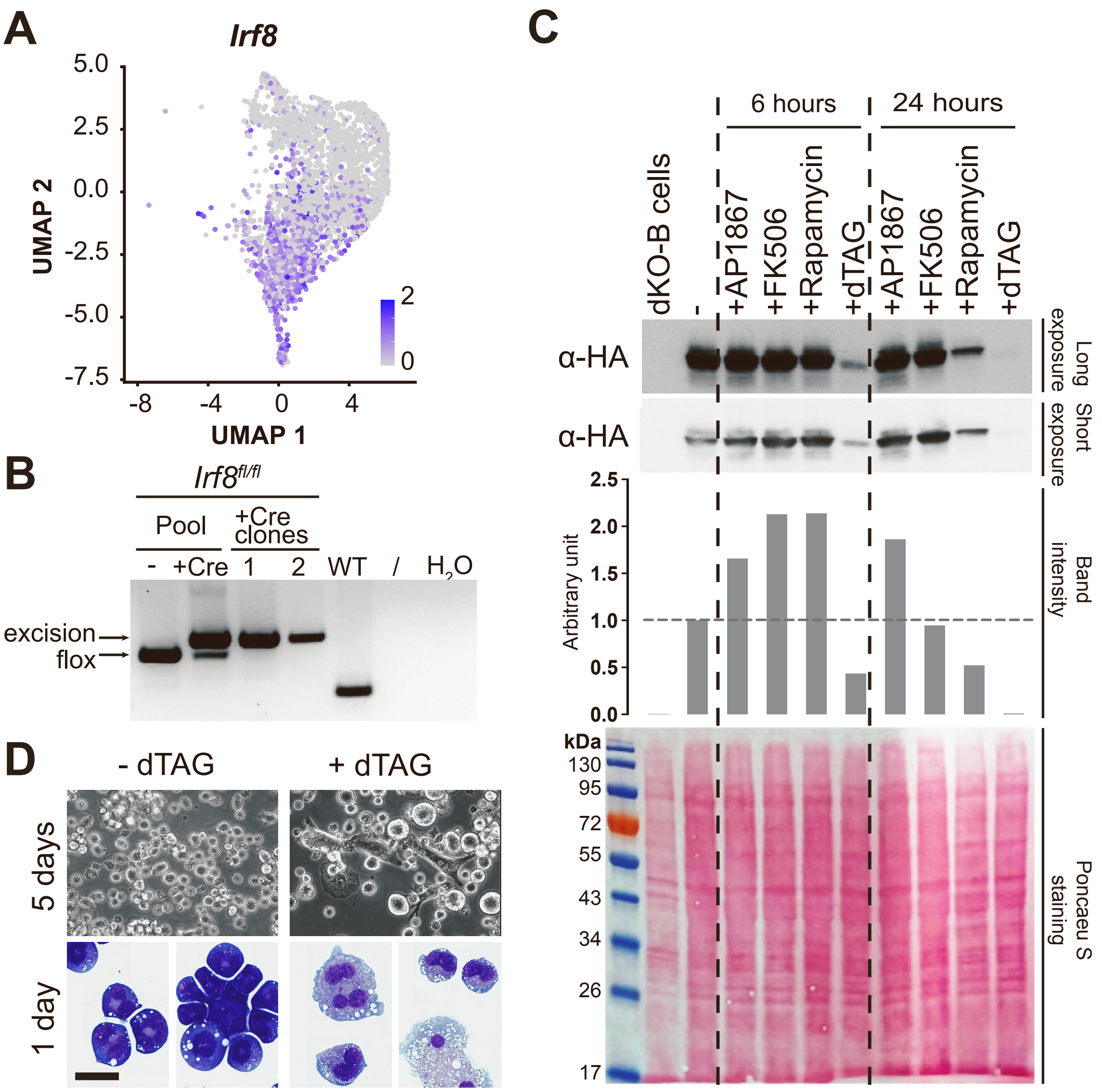

Table S1. (related to Figure 1D) Differentially expressed genes of myeloid clusters C1-C6. Genes with a corrected p-value <0.05 and FC >2 are shown.

Table S2. (related to Figure 1E) Enriched GO terms of biological processes in clusters C1-C6. Clusters marker genes with adjusted p-value <0.05 and FC >1.5 were used for this analysis.

SUPPLEMENTAL EXPERIMENTAL PROCEDURES

Vector constructs

The retroviral pMSCV-IRES-EGFP (MIEG) vector was used for ectopic C/EBPβ-LAP* expression as described previously (Cirovic et al., 2017). The C/EBPβ-LAP*-FKBP12^{F36V}-HA fusion expression construct was based on the rat C/EBPβ sequence (Gene ID #24253) and the FKBP12^{F36V}-HA was purchased from Addgene (#104371). The rat C/EBPβ-LAP* sequence was generated by PCR primers 5′-ccgctcgaggccaccatggcccaccgcctgctggcc and 3′-ctggcctcggcgggtcactgcggatccgcg from construct pMSCV-IRES-BFP C/EBPβ-LAP* (Cirovic *et al.*, 2017). FKBP12^{F36V} from pAW63.YY1.FKBP.knock-in.BFP was generated with the primers 5′-cgcggatccagcggtggaagtggtggcggagtgcaggtggaaacc and 3′-gatgtcccggactatgcataagaattccgg. Conditional STAT5A constructs were obtained from Richard Moriggl (University of Veterinary Medicine, Vienna, Austria) and were described previously (Grebien et al., 2008). Briefly, the STAT5 chimera was fused to the hormone-binding domain of the estrogen receptor (ER) variant to allow conditional activation by 4-hydroxytamoxifen (4-OH-T, Sigma-Aldrich). The GMPBT STAT5 cell lines were designated pMSCV-STAT5 WT, pMSCV-STAT5Δ749, or pMSCV-cS5.

Cells and differentiation

Cells with deletion of endogenous floxed *Cebpa* and *Cebpb* with TAT-Cre recombinase generated *Cebpa*$^{-/-}$*Cebpb*$^{-/-}$ cells (dKO-B cells). The B cells were cultured with 50 μM β-mercaptoethanol. Transdifferentiated GMPBT were selected by β-mercaptoethanol depletion as indicated previously (Cirovic *et al.*, 2017); this selection was applied in experiments involving CEBP-dKO-LAP*-FKBP12^{F36V}-GMPBT cells. After 2–3 weeks, only long-term proliferating transdifferentiated cells survived. *Irf8* knockout (KO) clones were generated from *Irf8*$^{fl/fl}$ v-Abl transformed B cells by incubation with TAT-Cre recombinase as described earlier. The Irf8 KO cell transdifferentiation was the same as that of the WT-B cells. Imatinib (Thermo Fischer Scientific; final concentration 0.4–0.6 μM), ruxolitinib (1 μM), and dTAG-13, FK506 (all, Tocris Bioscience), or AP1867 (ChemScene) (all, 1–5 μM) were added as indicated. CSF1, CSF2, and CSF3 were from PeproTech and added as indicated.

Antibody array

A pool of 10^8 GMPBT was harvested at day 32 p.i. (Experiment 1) or day 26 p.i. (Experiment 2). The harvested cells were washed and Fc-blocked using anti-mouse CD16/32 (TruStain FcX, BioLegend), then stained with a cocktail of fluorophore-conjugated anti-mouse antibodies against CD115 (AFS98, eBiosciences), Ly6G (1A8, BioLegend), and/or CD11b (M1/70, BD Pharmingen) for 20 minutes at 4°C. Lyophilized antibody plates containing specific phycoerythrin (PE)-conjugated antibodies were reconstituted following the manufacturer's instructions (LEGENDScreen™, BioLegend). The stained cells were distributed to individual wells in LEGENDScreen™ plates, washed and fixed according to the manufacturer's instructions before undergoing flow cytometric analysis using a MACSQuant analyzer (Miltenyi).

Two independent experiments were performed, with optimizations in Experiment 2, which included cell number reduction and CD11b staining exclusion (as 100% of the cells were CD11b$^+$). The measured data were processed with FlowJo following **Figure S1A** gating strategy**.** In each subset (CD115$^+$, Ly6G$^+$, Ly6G$^-$CD115$^-$ double-negative [DN]), the PE signal was overlaid against the PE signal of the equivalent isotype control to identify PE-positive percentages, which were used to compose surface antigen expression heatmaps. Negative or low-expression markers (sum PE positivity in all three subsets < 8% or <15% applied to Experiment 1 and 2, respectively) were excluded. As both experiments had similar marker expression patterns, we chose Experiment 2 for subsequent analysis.

Cytospin and Giemsa-May-Grünwald staining

Cells (1×10^4) were spun onto glass slides at 500 rpm for 5 minutes using a cytocentrifuge (Aerospray slide stainer, Wescor). The slides were air-dried, fixed with methanol for 5 minutes, immersed in May-Grünwald (Merck) solution for 5 minutes, washed with water, then immersed in Wright-Giemsa (Merck) solution for 45 minutes. After extensive rinsing in water, the slides were dried and cover-slipped (Roti-Histokitt II).

Bioinformatic analysis

Sequencing reads were demultiplexed and aligned with the mm10 genome using Cell Ranger 2.1.0 before further analyses using R 4.1.2. For the overview of the C/EBP family expression (Supplementary Figure S1E), an updated version of mm10 and CellRanger version 7.1 were used. The data was

preprocessed by the Cell Ranger pipeline 2.1.0 and analyzed using Seurat 4 (Satija et al., 2015). Raw data was filtered by removing cells with <400 features and >4000 features. Additionally, cells containing a mitochondrial read percentage of >7 were removed as potentially dead cells. The WT-B and dKO-B samples were normalized using the LogNormalize option, integrated, and scaled. Uniform manifold approximation and projection (UMAP) dimension reduction (McInnes, Healy and Melville, 2018) and clustering was performed by a shared nearest neighbor (SNN) modularity optimization-based clustering algorithm with the resolution parameter set to 0.5.

SingleR was used for automated identification and annotation of the resulting cell clusters (Aran et al., 2019). Based on the Immunological Genome Project (ImmGen) database, the clusters were assigned to cell types by comparing the expression of the cells of each cluster to the expression data deposited in ImmGen. For each cluster, marker genes were determined using MAST (Finak et al., 2015) with a corrected p-value of 0.05 and a fold change (FC) of 2. Only genes that were expressed in ≥20% in at least one group were considered.

To determine the specific cluster functions, gProfiler (Reimand et al., 2016) was applied to the marker genes of each cluster as indicated previously, except for the use of a 1.5 FC. Gene Ontology biological pathways (GO:BP) and Kyoto Encyclopedia of Genes and Genomes (KEGG) terms were used as a source and considered relevant if the corrected p-value (false discovery rate [FDR]) was <0.001.

Gene set variation analysis (GSVA) was used for targeted identification of specific functional and literature-known gene sets, i.e., their specific expression in subgroups of our cells (Hanzelmann, Castelo and Guinney, 2013), to several gene sets. Gene sets for Figure S2A,B are derived from Giladi et al. (Giladi et al., 2018) and gene sets for Figure S2I are derived from Weinreb et al. (Weinreb et al., 2020).

Developmental trajectories were inferred with Slingshot (Street et al., 2018) with cluster 1 as the starting point, as SingleR determined that it was the most precedent cluster.

To predict TFs involved in the onset of the granulocytic and monocytic developmental branch, we first identified the differentially expressed genes between clusters 1, 2 and 3 and the clusters 4, 5 and 6 (adjusted $p < 0.05$, $|FC| > 1.2$). We then applied LISA (Qin et al., 2020) to the set of up and down regulated genes in a comparative mode.

References

Aran, D., Looney, A.P., Liu, L., Wu, E., Fong, V., Hsu, A., Chak, S., Naikawadi, R.P., Wolters, P.J., Abate, A.R., Butte, A.J., and Bhattacharya, M. (2019). Reference-based analysis of lung single-cell sequencing reveals a transitional profibrotic macrophage. Nat Immunol *20*, 163-172. 10.1038/s41590-018-0276-y.

Cirovic, B., Schonheit, J., Kowenz-Leutz, E., Ivanovska, J., Klement, C., Pronina, N., Begay, V., and Leutz, A. (2017). C/EBP-Induced Transdifferentiation Reveals Granulocyte-Macrophage Precursor-like Plasticity of B Cells. Stem Cell Reports *8*, 346-359. 10.1016/j.stemcr.2016.12.015.

Finak, G., McDavid, A., Yajima, M., Deng, J., Gersuk, V., Shalek, A.K., Slichter, C.K., Miller, H.W., McElrath, M.J., Prlic, M., Linsley, P.S., and Gottardo, R. (2015). MAST: a flexible statistical framework for assessing transcriptional changes and characterizing heterogeneity in single-cell RNA sequencing data. Genome Biol *16*, 278. 10.1186/s13059-015-0844-5.

Giladi, A., Paul, F., Herzog, Y., Lubling, Y., Weiner, A., Yofe, I., Jaitin, D., Cabezas-Wallscheid, N., Dress, R., Ginhoux, F., et al. (2018). Single-cell characterization of haematopoietic progenitors and their trajectories in homeostasis and perturbed haematopoiesis. Nat Cell Biol *20*, 836-846. 10.1038/s41556-018-0121-4.

Grebien, F., Kerenyi, M.A., Kovacic, B., Kolbe, T., Becker, V., Dolznig, H., Pfeffer, K., Klingmuller, U., Muller, M., Beug, H., Mullner, E.W., and Moriggl, R. (2008). Stat5 activation enables erythropoiesis in the absence of EpoR and Jak2. Blood *111*, 4511-4522. 10.1182/blood-2007-07-102848.

Hanzelmann, S., Castelo, R., and Guinney, J. (2013). GSVA: gene set variation analysis for microarray and RNA-seq data. BMC Bioinformatics *14*, 7. 10.1186/1471-2105-14-7.

McInnes, L., Healy, J., and Melville, J. (2018). UMAP: Uniform Manifold Approximation and Projection for Dimension Reduction. 10.48550/arxiv.1802.03426.

Qin, Q., Fan, J., Zheng, R., Wan, C., Mei, S., Wu, Q., Sun, H., Brown, M., Zhang, J., Meyer, C.A., and Liu, X.S. (2020). Lisa: inferring transcriptional regulators through integrative modeling of public chromatin accessibility and ChIP-seq data. Genome Biol *21*, 32. 10.1186/s13059-020-1934-6.

Reimand, J., Arak, T., Adler, P., Kolberg, L., Reisberg, S., Peterson, H., and Vilo, J. (2016). g:Profiler-a web server for functional interpretation of gene lists (2016 update). Nucleic Acids Res *44*, W83-89. 10.1093/nar/gkw199.

Satija, R., Farrell, J.A., Gennert, D., Schier, A.F., and Regev, A. (2015). Spatial reconstruction of single-cell gene expression data. Nat Biotechnol *33*, 495-502. 10.1038/nbt.3192.

Street, K., Risso, D., Fletcher, R.B., Das, D., Ngai, J., Yosef, N., Purdom, E., and Dudoit, S. (2018). Slingshot: cell lineage and pseudotime inference for single-cell transcriptomics. BMC Genomics *19*, 477. 10.1186/s12864-018-4772-0.

Weinreb, C., Rodriguez-Fraticelli, A., Camargo, F.D., and Klein, A.M. (2020). Lineage tracing on transcriptional landscapes links state to fate during differentiation. Science *367*. 10.1126/science.aaw3381.
