# Supplementary material for: C/EBPβ-induced lymphoid-to-myeloid transdifferentiation emulates granulocyte-monocyte progenitor biology
Source: Stem Cell Reports. 2023 Dec 28;19(1):112–25. doi: 10.1016/j.stemcr.2023.11.011 (PMC10828814; doi:10.1016/j.stemcr.2023.11.011)
Supplement: Document S2. Article plus supplemental information [file mmc4.pdf]

Stem Cell Reports
Resource
OPEN ACCESS

C/EBPβ-induced lymphoid-to-myeloid transdifferentiation emulates granulocyte-monocyte progenitor biology

Linh Thuy Nguyen,[1,4,5] Karin Zimmermann,[1,5] Elisabeth Kowenz-Leutz,[1] Ramonique Lim,[1] Maria Hofstätter,[1] Alexander Mildner,[1,2,3] and Achim Leutz[1,*]
[1]Max Delbrück Center for Molecular Medicine in the Helmholtz Association, Robert-Roessle-Str. 10, Berlin, Germany
[2]Institute of Biomedicine at University of Turku, Turku, Finland
[3]InFLAMES Research Flagship, University of Turku, 20014 Turku, Finland
[4]Berlin School of Integrative Oncology (BSIO), Charité – Universitätsmedizin Berlin, Corporate Member of Freie Universität Berlin and Humboldt-Universität zu Berlin, Berlin, Germany
[5]These authors contributed equally
*Correspondence: aleutz@mdc-berlin.de
https://doi.org/10.1016/j.stemcr.2023.11.011

SUMMARY

CCAAT/enhancer-binding protein beta (C/EBPβ) induces primary v-Abl immortalized mouse B cells to transdifferentiate (BT, B cell transdifferentiation) into granulocyte-macrophage progenitor-like cells (GMPBTs). GMPBTs maintain cytokine-independent self-renewal, lineage choice, and multilineage differentiation. Single-cell transcriptomics demonstrated that GMPBTs comprise a continuum of myelomonopoietic differentiation states that seamlessly fit into state-to-fate maps of normal granulocyte-macrophage progenitors (GMPs). Inactivating v-Abl kinase revealed the dependence on activated CSF2-JAK2-STAT5 signaling. Deleting IRF8 diminished monopoiesis and enhanced granulopoiesis while removing C/EBPβ-abrogated self-renewal and granulopoiesis but permitted macrophage differentiation. The GMPBT culture system is easily scalable to explore the basics of GMP biology and lineage commitment and largely reduces ethically and legislatively debatable, labor-intensive, and costly animal experiments.

INTRODUCTION

The classic view of homeostatic hematopoiesis emerged from studies that used transplantation, developmental cell tracing, and colony formation assays. According to this classic view, hematopoietic stem cells (HSCs) are the only blood cells that can self-renew and give rise to progenitors with progressively restricted differentiation potential following linear bifurcating cell lineage decisions. This hierarchy is typically shown as an upside-down decision tree diagram with self-renewing HSCs at the top giving rise to multipotent progenitors (MPPs) with limited self-renewal capacity. As these progenitors become increasingly specialized, they differentiate into common myeloid progenitors and common lymphoid progenitors (CMPs and CLPs, respectively). CMPs subsequently branch into megakaryocyte-erythroid progenitors (MEPs) and granulocyte-macrophage progenitors (GMPs), which are the primary source of innate immune cells (Iwasaki and Akashi, 2007).

Recent advancements in single-cell RNA (scRNA) expression profiling, chromatin accessibility studies, and barcoded lineage tracing have questioned the strictly binary, unidirectional processes of hematopoietic cell fate decisions suggested by the hierarchical model. Instead, it appears that progenitor cells can be committed to a specific fate at an earlier stage of differentiation and, in addition, that progenitors at different stages may display developmental plasticity and are capable to switch lineages under certain circumstances (Naik et al., 2013). These characteristics have implications for the production of innate immune cells (namely, granulocytes and various types of monocytes) and may play a role in lineage infidelity or ambiguity, as observed in myelodysplasia and leukemia (Dress et al., 2020; Pucella et al., 2020).

Alternative innate immune cell fate decisions occur during the GMP state, such as granulocytic vs. monocytic differentiation (Liu et al., 2019). GMPs also have transit-amplifying capacity and are vulnerable to leukemia initiation, which may lead to the development of self-renewing leukemic stem cells (LSCs) that perpetuate the disease (Krivtsov et al., 2006; Ye et al., 2015). Therefore, understanding the biology of GMPs is essential for comprehensively understanding specification of innate immune cells in homeostasis, regeneration, and leukemogenesis (Herault et al., 2017; Niederkorn and Starczynowski, 2017).

Experimental manipulation of hematopoietic lineage identity, such as by ectopic expression of lineage-defining master or pioneering transcription factors (TFs), can reveal the plasticity and decision-making processes involved (Graf and Enver, 2009; Regalo and Leutz, 2013). For example, primary mouse pre-B cells that were transformed by the v-Abl oncogene could be transdifferentiated into a GMP-like population (GMPBTs) by ectopic expression of the TF CCAAT/enhancer-binding protein beta (C/EBPβ)-LAP* (Cirovic et al., 2017). In contrast to HoxA8/A9-induced precursor cells, C/EBPβ-LAP*-derived GMPBTs maintain a self-renewing progenitor population and continuously undergo spontaneous lineage choice to produce both neutrophils

This is an open access article under the CC BY license (http://creativecommons.org/licenses/by/4.0/).

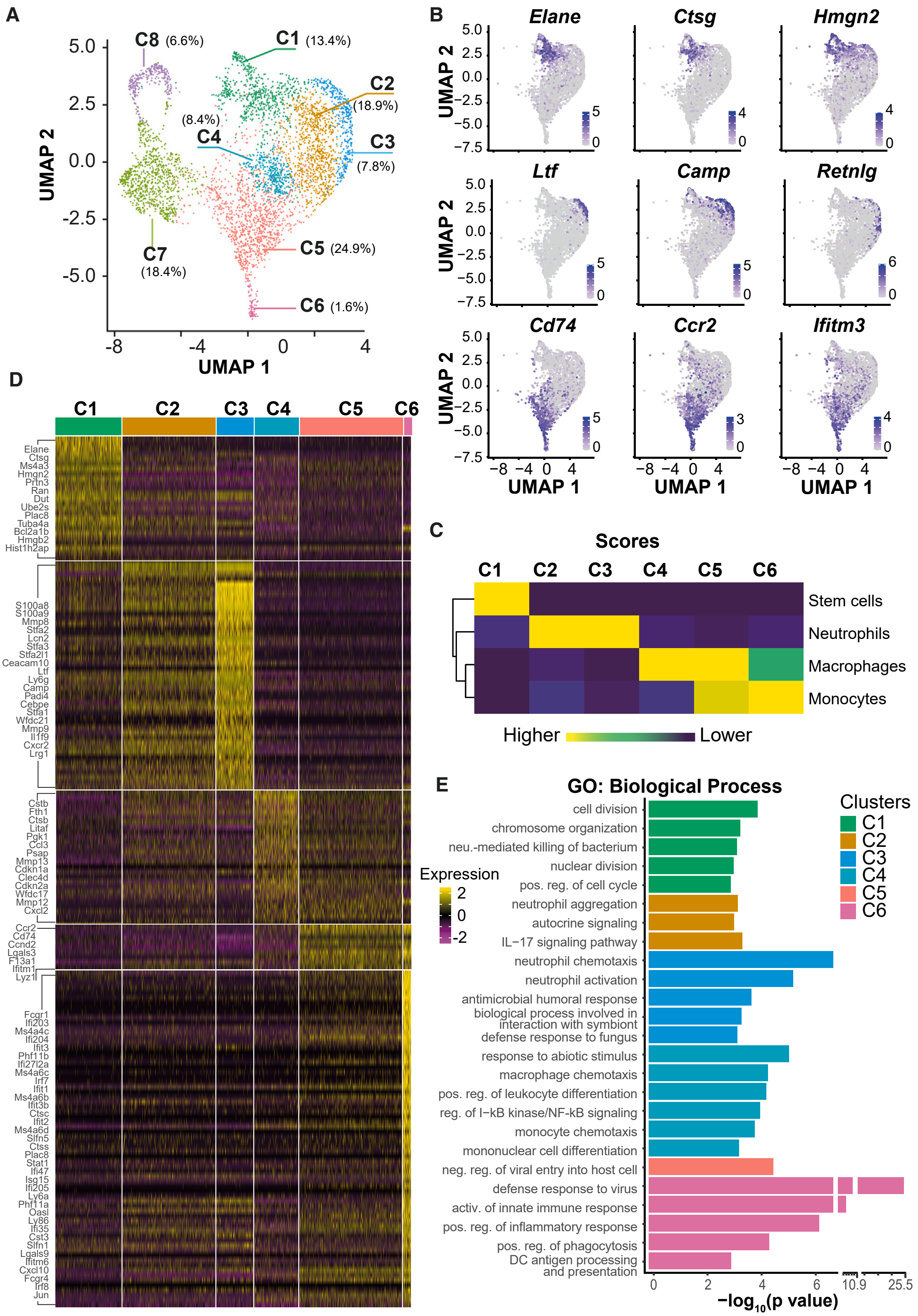

(legend on next page)

(Ly6G⁺/CD115⁻) and monocytes (Ly6G⁻/CD115⁺). Accordingly, GMPBTs exhibit similarities to both GMPs and LSCs, as observed in chronic myelogenous leukemia (CML; as a product of BCR-ABL translocation).

Here, we characterized GMPBTs using scRNA sequencing (scRNA-seq) and cell biological and molecular genetic analyses to investigate the biology and lineage decision processes. We demonstrate that GMPBTs largely resemble normal *ex vivo*-isolated GMPs and that GMPBT maintenance relied on v-Abl-mediated signaling involving C/EBPβ and the signal transducer and activator of transcription 5A (STAT5). GMPBTs could be expanded to large quantities that were amenable to molecular genetics, biochemical, pharmacological, and cell biological experimentation. GMPBTs therefore serve as a valuable model system to experimentally explore basic processes involved in myelomonocytic lineage commitment and cell differentiation and also reduce the need for animal experimentation.

RESULTS

Myelomonocytic differentiation potential of GMPBTs

Previously, we have demonstrated that C/EBPβ-LAP* lymphoid-myeloid lineage-switched GMPBTs maintained an immature state and continuously produced granulocytes and monocytes/macrophages (Cirovic et al., 2017). Here, we characterized GMPBTs with droplet-based scRNA-seq. Briefly, GMPBTs were generated from wild-type (WT) B cells by retroviral infection with a pMSCV-IRES-EGFP-C/EBPβ-LAP* construct. Sorted GFP-positive (GFP⁺) cells were examined on day 6 post infection (p.i.) by scRNA-seq. A pure population of GMPBTs can be acquired through the exclusion of β-mercaptoethanol, which effectively eliminates residual B cells. Nevertheless, we intentionally omitted this selection process to accommodate the inclusion of lymphoid-myeloid transition states in our scRNA-seq analysis. At harvesting, surface marker analysis revealed that the GFP⁺ cell pool contained 72.6% transdifferentiated CD11b⁺ cells and 11.1% un-transdifferentiated CD19⁺ cells. The CD11b⁺ cell fraction contained subpopulations of granulocytic Ly6G⁺ (4.9%), monocytic CD115⁺ (60%), and Ly6G⁻ CD115⁻ double-negative (DN) cells (30.9%) (Figure S1A).

scRNA-seq reads were obtained from 3,297 cells with a median of 7,799 unique molecular identifier (UMI) counts and 1,763 genes per cell. Dimensional reduction of the dataset and subsequent clustering yielded eight distinct clusters (Figure 1A). Cells in clusters 7 and 8 mainly expressed B lymphoid-specific genes (*Ebf1*, *Vpreb1/2/3*, and *CD79a*) and, in accordance with the remaining CD19⁺ population, were defined as B cells (Figure S1B). Cluster 7 was additionally enriched for genes related to cell cycle and cell division (*Top2a*, *Hmgnb1/2*, and nucleosome proteins *Hist1h2ap* and *H2afx*) (Figure S1B).

For further analysis, we concentrated on clusters 1–6, which expressed typical myeloid lineage genes (Figure 1B). Genes that define the neutrophil lineage were enriched in clusters 1, 2, and 3 (*Ltf*, *Camp*, *Elane*, and *Ctsg*), whereas clusters 4, 5, and 6 expressed genes characteristic for the monocyte/macrophage lineage (*Cd74*, *Ccr2*, and *Ifitm3*) (Figure 1B). To determine the cell identity of the clusters, we applied the automated cell type annotation tool SingleR with the ImmGen database as a reference (Heng et al., 2008). As shown in Figure 1C, cluster 1 was identified as stem cells despite the simultaneous expression of early neutrophil genes, which suggested that cluster 1 contained mostly myelomonocytic progenitors.

Further inspection of clusters 1–3 revealed that cluster 1 highly expressed the early granulocyte markers *Elane*, *Ctsg*, and *Prtn3* (Giladi et al., 2018) in addition to the GMP-defining *Ms4a3* (Figure 1D; Liu et al., 2019). Enhanced expression of cell cycle regulators in cluster 1 supported its progenitor characteristics. Cluster 3 was enriched for mature neutrophil markers (*Ly6g*, *S100a8/9*, and *Cebpe*) and several signaling proteins involved in immune responses (*Stfa1/2/3*, *Lcn2*, and *Wfdc21*). Cluster 2 did not have a unique gene expression pattern but demonstrated

Figure 1. Granulocyte and macrophage lineage capacity of GMPBTs
(A) WT B cells were retrovirally transduced with *Cebpb*-LAP*. 6 days post infection (p.i.), cells were subjected to scRNA-seq. Dimension reduction (uniform manifold approximation and projection, UMAP) and clustering of 3,297 cells identified 8 clusters. Percentages of cells in clusters are indicated.
(B) Feature plots of myeloid gene expression. Neu-specific genes are expressed in 3 different clusters (cluster 1: *Elane*, *Ctsg*, and *Hmgn2*; clusters 2 and 3: *Ltf*, *Camp*, and *Retnlg*). Monocyte/macrophage-specific genes are expressed in clusters 4, 5, and 6 (*Cd74*, *Ccr2*, and *Ifitm3*).
(C) Cell type annotation of clusters 1–6 using the SingleR and ImmGen databases as reference. The annotation with the highest score is shown for each cluster.
(D) Heatmap of differentially expressed marker genes (rows) of myeloid clusters 1–6 (columns). Genes with corrected $p < 0.05$ and $|FC|$ (fold change) > 2 are shown in the heatmap; representative genes are listed on the left. A table of all genes contained in the heatmap is provided in Table S1.
(E) Gene ontology (GO) enrichment for marker genes ($|FC| > 1.5$, adjusted $p < 0.05$) of myeloid clusters 1–6 using gProfiler. GO terms of "biological processes" are shown; redundant terms were excluded. For a complete list of GO terms enriched in each cluster, see Table S2. Color code as indicated in (A) and (D).

diminished expression of cluster 3-specific genes and limited expression of some cluster 4 genes. We therefore considered cluster 2 a transitory population between clusters 1 and 3 or 4. Functional analysis of the cluster markers (clusters 1–3, |FC| >1.5, adjusted $p < 0.05$) using gProfiler (Reimand et al., 2016) revealed enrichment for terms related to cell cycle and division in cluster 1, while terms associated with neutrophil activities were annotated to clusters 2 and 3 (Figure 1E).

Both clusters 5 and 6 were enriched for monocytic genes, including the major histocompatibility complex (MHC) class II-related gene *Cd74*, the chemokine receptor *Ccr2*, the macrophage colony-stimulating factor (M-CSF) receptor *Csf1r*, the lysosomal protease *Ctsc*, and lysozyme 1 (Figure 1D). Cluster 6 contained higher expression of the key differentiation TF *Irf8*, inflammatory-responsive TF *Stat1*, the surface marker *Fcgr1* (CD64), and the interferon-inducible *Ifi* gene family together with enrichment of immune response Gene Ontology (GO) terms (innate immune response, defense response to virus, and phagocytosis), further indicating monocyte/macrophage identity (Yanez et al., 2017). Cluster 4 linked the neutrophil clusters 1–3 and the monocyte/macrophage clusters 5 and 6. Besides low expression of the monocyte-specific genes *Cd14* and *Fcgr3* (encoding CD16), cluster 4 also expressed genes related to lysosomal activity (*Lamp1*, *Cd68*, and *Psap*), enzymes (*Pkg1*, *Pgam1*, and *Gpi1*), and iron transporters (*Fth1* and *Ftl1*), indicating the phagocytic potential of these cells. We considered cluster 4 a transitory population undergoing monocyte/macrophage specification. Altogether, the GMPBT population contained cells in transitioning stages of granulocytic/neutrophil (G) and monocytic/macrophage (M) differentiation (Figures 1C—1E), suggesting that GMPBTs continuously undergo lineage choice and differentiation toward G/M fates, reflecting normal GMPs or LSCs (the latter because of the transforming activity of the v-Abl oncogene).

The endogenous *Cebpa* and *Cebpb* genes cross-regulate each other and can be induced by ectopic expression of either C/EBP TF. Therefore, we explored the C/EBPβ-LAP*-induced transdifferentiation outcome in the absence of endogenous C/EBPα and C/EBPβ. We compared GMPBTs derived from either WT B cells or double knockout (dKO) B cells by scRNA-seq (Figures S1A and S1C). A total of 1,423 dKO B cells were recovered for the analysis, with a median of 7,975 UMIs and 1,914 genes per cell. Data from the WT B cell and dKO B cell pools were integrated before clustering, and all 8 clusters were present in both samples, suggesting similar cell type outcomes in both genotypes (Figure S1D), although quantitative differences were also noted. B lymphoid clusters 7 and 8 represented 33% of the dKO B cells and 11% of WT B cells, respectively, coinciding with the respective CD19 marker expression in the cells. In contrast, cluster 1–6 WT B cells featured more myelomonocytic cells (72.6% $CD11b^+$) compared with dKO B cells (38.2% $CD11b^+$).

We conducted a comprehensive assessment of the gene expression of the C/EBP family members, encompassing C/EBPα, C/EBPβ, C/EBPδ, C/EBPε, C/EBPγ, and C/EBPζ within myeloid clusters 1–6 (Figure S1E). Upon ectopic expression of C/EBPβ LAP*, when endogenous *Cebpa* and *Cebpb* were absent, a significant reduction of C/EBPδ and C/EBPε expression was observed, while C/EBPγ and C/EBPζ remained largely unaffected. This finding suggested that C/EBPα, either independently or in conjunction with C/EBPβ, plays a regulatory role in the modulation of C/EBPε and C/EBPδ expression. The absence of C/EBPδ in all clusters of the dKO GMPBTs indicates a dependence on regulation by C/EBPα,β and raises the possibility of a previously unexplored role of C/EBPδ in myelopoiesis. Collectively, these outcomes underscore that, while GMPBT transdifferentiation occurs even in the absence of endogenous C/EBPα,β, the intricate mutual cross-regulation of the C/EBP family could substantially contribute to the efficiency and configuration of the transdifferentiation process.

The GMPBT transcriptome reflects the fate-transitioning landscape of GMPs

We compared the GMPBT scRNA-seq data with published precursor gene expression profiles to explore the occurrence of cell types and differentiation stages of GMPBTs. Hematopoietic differentiation inferred from snapshot single-cell analysis (Giladi et al., 2018) and dynamic state-to-fate maps derived from clonally tagged stem/progenitors (lineage and RNA recovery [LARRY] method) (Weinreb et al., 2020) were compared with GMPBT scRNA-seq data (Figures 2 and S2). GMPBT clusters 2 and 3 coincided with neutrophils and clusters 5 and 6 with monocytes/macrophages matching with corresponding *in vivo* lineage signatures defined by Giladi et al. (2018) (Figures S2A and S2B). The vast majority of the GMPBTs also merged seamlessly into the *ex vivo* mouse bone marrow expression profile (hereafter called LARRY profile) (Figure 2A) and interleaved all LARRY-defined neutrophil and monocyte clusters. The GMPBT population lacked several cell types identified by LARRY, including $CD34^+$ MPPs and cells that belong to the $Gata2^+$ erythroid/megakaryocyte/mast/basophil/eosinophil fate. These data were in accordance with the notion that GMPBTs resemble GMPs rather than other earlier progenitor stages. The GMPBT data projection (Figure 2A) also revealed direct connections between the remaining B cell cluster (expressing *Pax5* and *Ebf1*) and monocyte/macrophage clusters (expressing pan-myeloid markers *Spi1* and *Lyz2*), which suggested an additional transdifferentiation trajectory directly into monocytes/macrophages (Figures 2A and 2B).

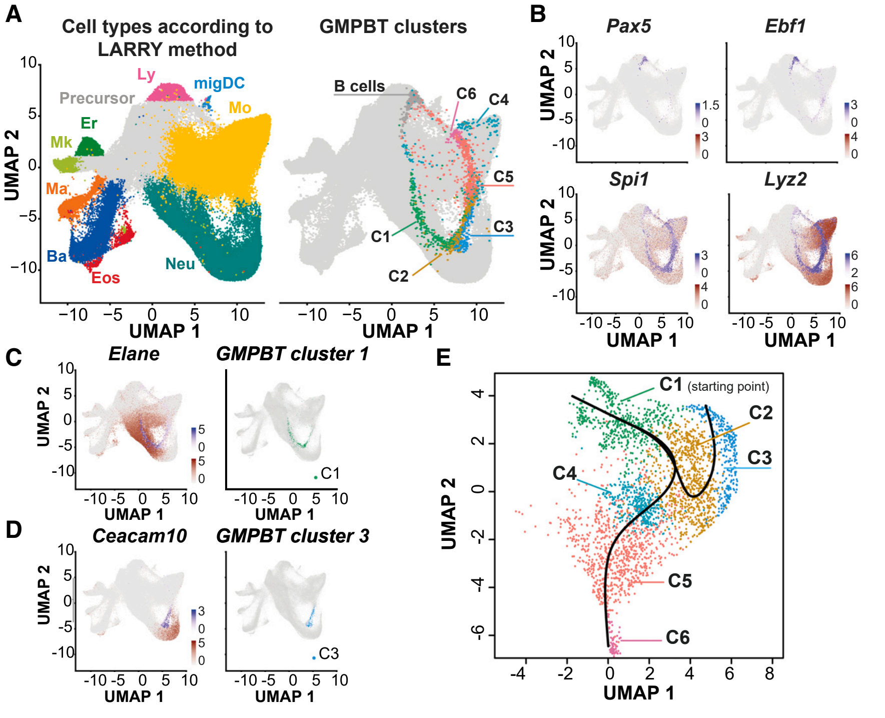

Figure 2. The GMPBT transcriptome reflects the fate-transitioning landscape of GMP differentiation

(A) Integration of GMPBT scRNA profiling data with lineage-traced mouse bone marrow cell data according to Weinreb et al. (2020). scRNA-seq data from LARRY-traced mouse bone marrow were used as a reference; the GMPBT data were projected as a query. Cell types identified in the reference are indicated by color (left). The UMAP of integrated data is shown on the right, with reference data shown in gray and GMPBT clusters overlayed. Color of GMPBT clusters as in Figure 1A.

(B) Expression of *Pax5 and Ebf1*, marking B cell clusters (top), and expression of *Spi1 and Lyz2*, marking myeloid clusters (bottom). The expression of these genes in the reference dataset and in the GMPBTs is presented in brown and purple, respectively.

(C and D) Expression of *Elane* and *Ceacam10* in the reference dataset (brown) and GMPBTs (purple) is shown as merged plots on the left. Shown on the right are GMPBT cluster 1 and cluster 3.

(E) Trajectory analysis of the myeloid clusters 1–6 using Slingshot. Based on the expression of proliferation genes, cluster 1 (C1) was designated as the starting point. Two differentiation trajectories are shown.

The neutrophil markers *Elane* and *Ceacam10* mark distinct groups of cells in a continuous landscape as defined by the LARRY profile (Figures 2C and 2D). *Elane*-expressing cells were previously considered precursors of *Ceacam10*-expressing cells (Giladi et al., 2018). We detected an overlapping distribution between LARRY *Elane*-expressing cells with *Elane*-expressing GMPBTs in cluster 1 (Figure 2C). Similarly, we identified co-alignment of LARRY *Ceacam10*-expressing mature neutrophils with *Ceacam10*-expressing GMPBTs (cluster 3; Figure 2D), which suggested that the GMPBT system recapitulated sequential transition stages of granulopoietic differentiation.

The current findings indicated that GMPs and neutrophil precursors in mammals entail proliferation capacity (transit-amplifying cells) (Evrard et al., 2018). During granulopoiesis, GMP transit through a CD106$^+$ (*Vcam1*) proneutrophil stage before differentiating into mature neutrophils (Kwok et al., 2020). Similarly, our dataset presented the highest expression of *Vcam1* in cluster 1 (Figure S2C). Analysis of granulopoiesis-essential TFs demonstrated that cluster 1 also contained the highest expression of *Cebpa* among the three neutrophil clusters, known for initiating myeloid GMP differentiation, together with *Runx1* and the granulopoiesis-directing factor *Gfi1* (Figure S2D). *Irf8* is expressed and active at similar levels in both granulocytic and monocytic precursors but not in GMPs (Yanez et al., 2015). Expression of *Irf8* in cluster 2 supported the idea that this cluster likely represents an intermediate stage in the neutrophil differentiation hierarchy. Finally, cluster 3 expressed TFs involved in terminal neutrophil maturation and function, such as *Spi1* and *Cebpe* (Giladi et al., 2018). Furthermore, the expression of genes related to granule production, chemotaxis, and phagocytosis aligned with this description (Figures S2E–S2G).

GMPBT monocyte clusters 4, 5, and 6 overlapped with both the LARRY monocyte branch (Figure 2A) and the monocyte-defining gene set of Giladi et al. (2018). Many monocyte-restricted genes were highly expressed in clusters 5 and 6, whereas cluster 4 showed the least correlation to the mature monocyte signature (Mildner et al., 2017; Figure S2B). Cluster 4 cells demonstrated residual expression of proliferation-related genes, including *Cdk2*, *Cdk4*, and *Ccnb2*, and expressed the highest levels of *Cebpb* and *Nr4a1* compared with the other monocytic clusters (Figure S2H). Based on this expression pattern, we considered cluster 4 monocyte progenitors. Cluster 5 cells exhibited more similarities to monocyte progenitors in cluster 4 but also expressed *Cd74*, *Ccr2*, *Lgals3*, and *F13a1*. Cluster 6 cells showed more similarities to Immgen monocytes (Figure 1C), including the expression of *Plac8*, *Fcgr1*, and *Ms4a6c*, but in addition expressed interferon type I-inducible genes; e.g., *Ifit3*, *Isg15*, *Ifi204*, and *Oasl*.

The concept of two alternative monocyte differentiation pathways into dendritic cell (DC)-like or neutrophil

(Neu)-like monocytes has been suggested previously based on the LARRY profile (Weinreb et al., 2020). We therefore compared GMPBT clusters 4, 5, and 6 for DC-like or Neu-like macrophage signatures derived from the LARRY dataset. The distribution of GMPBTs with DC- or Neu-like signatures could be distinguished (Figure S2I), and DC-like cells were mostly found in cluster 6, likely because of their high MHC class II- and interferon type I-related gene expression pattern, while the Neu-like monocyte signature was enriched in cluster 5, which is more related to Neu cluster 3.

Finally, the developmental relationships among all six clusters were predicted with the Slingshot trajectory analysis (Street et al., 2018; Figure 2E). Cluster 1 was set as the starting point based on the high expression of proliferation genes. The trajectory analysis revealed bifurcation in cluster 2, branching into mature Neus (cluster 3) and monocyte progenitor cells (cluster 4). Subsequent differentiation prediction included Neu maturation (cluster 2 to cluster 3) and the differentiation of mature monocytes/macrophages with DC similarities (cluster 6).

Taken together, integration of data derived from the GMPBT scRNA-seq transdifferentiation profile, *in vivo* transcriptome, and *ex vivo* LARRY myelopoiesis fate maps suggested that GMPBTs recapitulated normal granulocytic-monocytic commitment and represent a continuum of intermediary cell fate specification steps toward Neus and monocytes/macrophages. The GMPBT system therefore largely mirrors normal GMP biology and may be useful to examine mechanisms involved in cell fate choice.

GMPBT surface markers

The cell lineage fidelity of WT GMPBT was next examined by flow cytometry screening of surface marker expression using an antibody array of 255 phycoerythrin (PE)-conjugated antibodies (LEGENDScreen, BioLegend). The bipotential differentiation capacity of GMPBTs into Neus and monocytes was confirmed by co-staining with CD11b, Ly6G, and CD115. The pan-hematopoietic or pan-myeloid markers (CD44, CD45, CD11b, and CD371) were present on all three GMPBT subsets (Ly6G$^-$CD115$^-$ DN progenitor population, Ly6G$^+$CD115$^-$ Neus, and Ly6G$^-$CD115$^+$ monocytes/macrophages) (Figure S2J). All cells also expressed CD62L, indicating the restriction of GMP potential to myeloid progenitors (Ito et al., 2021). The DN fraction contained higher expression of many markers representing undifferentiated stem/progenitor cells; e.g., CD51, CD106, Notch2, and CD31. The Ly6G$^-$CD115$^+$ subset was enriched for the expression of monocyte/macrophage markers (CD14, Mac-2, F4/80, and CD200R). Markers representing DC (Toll-like receptor [TLR] homolog CD180, TLR4, H-2, and CD275) were also highly expressed in this subset, which confirmed the differentiation potential toward DC-like cells. The Ly6G$^+$CD115$^-$ subset expressed the Neu-specific markers CD182 or plasmacytoid DC-specific triggering receptor expressed on myeloid cells (PDC-TREM) and epithelial tissue signatures (CD55, CD146, and CD100). Notably, certain markers associated with other cellular lineages were also detected. For instance, the DN subset displayed the presence of CD71, an indicator of erythroid lineage; the CD115$^+$ subset exhibited CD107a, a marker for activated natural killer cells; and the Ly6G$^+$ subset showed CD61, a marker indicative of megakaryocytes.

To establish a linkage between data obtained from both scRNA-seq and flow cytometric array screening, we aimed to isolate early Neu progenitors within the GMP fraction, residing in cluster 1 (Kwok et al., 2020). In this context, we identified CD106 (*Vcam1*) as a surface marker with pro-Neu specificity, expressed in the GMPBT DN fraction according to the LEGENDScreen results (Figures S2J and S2L). Intriguingly, CD106 RNA expression was almost exclusively observed in cluster 1 cells (Figures 1D and S2K). Subsequently, we sorted DN undifferentiated cells based on CD106 expression (DN-CD106$^+$) and compared them with DN-CD106$^-$ cells. While there was no marked distinction in the growth rate between these two populations (Figure S2M), the DN-CD106$^+$ cells displayed a higher proportion of Ly6G$^+$ Neus post sorting (Figure S2N), whereas differentiation into CD115 monocytes was roughly equivalent between DN-CD106$^+$ and DN-CD106$^-$ cells. These observations collectively indicated that DN-CD106$^+$ cells possess an elevated potential for neutrophilic differentiation, aligning with recent research findings (Kwok et al., 2020).

IRF8 and C/EBPβ expression levels determine GMPBT lineage fates

To identify potential regulators involved in the formation of the two trajectories observed in the GMPBT scRNA-seq data, we extracted genes that were differentially expressed between Neu clusters 1, 2, and 3 and compared them with monocyte/macrophage clusters 4, 5, and 6 (adjusted $p < 0.05$, $|FC| > 1.2$). Next, we predicted the most important TF involved in the differential regulation of these genes using LISA (epigenetic landscape *in silico* deletion analysis, Qin et al., 2020). As shown in Figure 3A, LISA highlighted enriched motives for IRF4, IRF8, and STAT within genes specifically expressed within the monocyte branch, while C/EBP and the erythroblast transformation specific TF family (ETS; ERG and FLI1) TF motives were enriched in genes of the granulocytic branch, respectively. Interestingly, motif enrichment of the master regulator of myeloid cells, PU.1, was predicted for both developmental branches.

Next, we explored how GMPBT biology is affected by intervention with lineage-defining TFs of critical GMP coregulatory complexes (CoRCs) (Arendt et al., 2016). IRF8 is part of a CoRC that supports macrophage differentiation

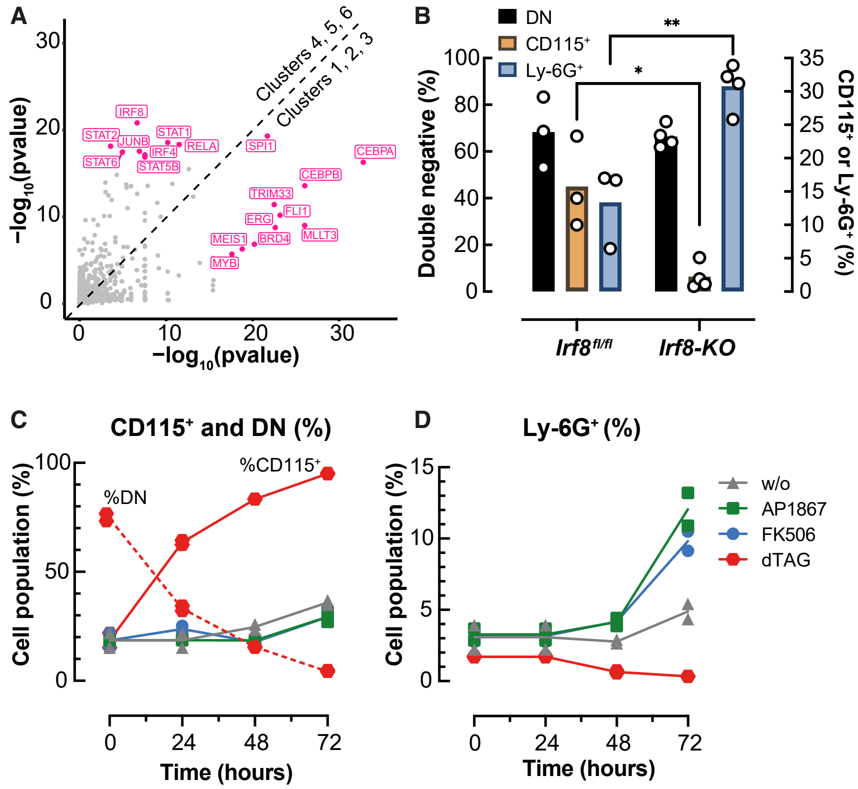

Figure 3. Function of IRF8 and C/EBP-β-LAP* on GMPBT subpopulations

(A) TF analysis using LISA on differentially expressed genes between two groups: Neu C1, C2, and C3 and monocyte/macrophage C4, C5, and C6 (adjusted $p < 0.05$, $|FC| > 1.2$).

(B) Comparison of transdifferentiated *Irf8*$^{fl/fl}$ and *Irf8* KO B cells. *Irf8* KO clones ($n = 4$) and isogenic *Irf8*$^{fl/fl}$ control clones ($n = 3$) were examined after retroviral expression of *Cebpb*-LAP*. Cells were subjected to flow cytometry analysis using antibodies directed to CD11b, Ly6G, and CD115. Flow cytometry parameters were gated as in Figure S1A. The graph shows percentages of the 3 GMPBT subpopulations: GMP-like cells (Ly6G^{-}CD115^{-}, double negative [DN], black bars, left y axis), granulocytes/Neus (Ly6G^{+}, orange bars, right y axis), and monocytes/macrophages (CD115^{+}, blue bars, right y axis). Data are mean ± SD from independent experiments, unpaired t tests, $^{**}p < 0.01$, $^{*}p < 0.05$; insignificance is not indicated.

(C) De-stabilization of LAP*-FKBP12^{F36V} by dTAG increased CD115^{+} cells and abrogated DN cells in GMPBT cultures. Shown are the kinetics and distribution of the CD115^{+} population (red line) and the CD115^{-}/Ly6G^{-} population (DN, red dashed line) of GMPBTs upon targeted proteolysis of LAP*-FKBP12^{F36V} by dTAG-13. Note that CD115^{+} monocytes increase from approximately 15% to 95%, while DN cells disappear during dTAG treatment. No effects on the CD115^{+} population are seen by treatment with FK506 or AP1867. Flow cytometry analysis was performed at 4 time points as indicated ($n = 2$, duplicates are shown, gating as shown in Figure S1A).

(D) Stabilization of LAP*-FKBP12^{F36V} by AP1867 or FK506 leads to an increase in Ly6G^{+} cells in GMPBT cultures (from approximately 3% to 9%–13%), while dTAG abrogated Ly6G^{+} cells. Flow cytometry analysis was performed at 4 time points as indicated ($n = 2$, duplicates are shown, gating as shown in Figure S1A).

The experiments shown in (C) and (D) were done in parallel, starting with the same GMPBT cultures. Drug treatments are shown on the right. No treatment, gray triangles; AP1867 treatment, green squares; FK506 treatment, blue dots; dTAG treatment, red dots.

from GMPs and impedes Neu differentiation. Removing *Irf8* from mice decreased monocyte numbers and caused neutrophilia that closely resembled CML (Holtschke et al., 1996; Tamura et al., 2015). We detected *Irf8* gene expression in the monocytic clusters 4, 5, and 6 and residual *Irf8* expression in cluster 2 (Figure S3A) that demarcated monopoiesis and granulopoiesis. To examine whether removing *Irf8* would skew GMPBT fates *in vitro*, we generated v-Abl-transformed B cells from a mouse strain with conditional *Irf8*$^{fl/fl}$ alleles (Feng et al., 2011). The *Irf8*$^{fl/fl}$ B cells were treated with a cell-permeable TAT-Cre recombinase to generate biallelic *Irf8*$^{-/-}$ clones (*Irf8* KO) or mock treated to generate isogenic controls (Figure S3B). By introducing LAP*, these cell clones were transdifferentiated into GMPBT-*Irf8*$^{fl/fl}$ or GMPBT-*Irf8* KO cells. GMPBTs from four independently derived *Irf8* KO clones and three *Irf8*$^{fl/fl}$ clones were examined for the expression of monocytic CD115 or granulocytic Ly6G surface markers (Figure 3B). The GMPBT-*Irf8* KO cells had a strong reduction of CD115^{+} monocyte/macrophages (>4-fold) and an approximately 3-fold increase of Ly6G^{+} Neus compared with the isogenic *Irf8*$^{fl/fl}$ controls. Monocytes/macrophages, but no granulocytes, were observed when C/EBPα was used to induce transdifferentiation of primary B cells, various B cell lines, or v-Abl-transformed B cells, although several Neu genes are initially activated during transdifferentiation (Bussmann et al., 2009; Xie et al., 2004). To explore the possibility that the remarkable failure of C/EBPα to induce granulopoietic transdifferentiation was extinguished by *Irf8* (Kurotaki et al., 2014), we modified this experiment using C/EBPα instead of C/EBPβ-LAP* in *Irf8* KO B cells. However, although macrophage transdifferentiation was recapitulated, no granulocyte differentiation was observed, which suggested that *Irf8* did not prevent Neu differentiation downstream of C/EBPα (data not shown). We concluded that the GMPBTs reflected IRF8-dependent

lineage choice, as observed in normal progenitors (Kurotaki et al., 2018; Paul et al., 2015).

Previously, we reported that GMPBT differentiation toward Ly6G$^+$ Neus is correlated with the magnitude of LAP* expression (Cirovic et al., 2017). To examine the importance of continuous C/EBPβ-LAP* expression after the completion of transdifferentiation, we constructed a proteolysis-targeting (PROTAC) chimera (LAP*-FKBP12^{F36V}) to conditionally remove C/EBPβ-LAP* from the transdifferentiated GMPBTs. Briefly, FKBP12^{F36V} chimeras are targeted to proteolysis by the non-toxic heterobifunctional degradation tag (dTAG)-13 compound that binds to the FKBP12^{F36V} moiety and recruits the CRBN E3 ligase, causing proximity-induced ubiquitinylation and proteasomal degradation (Nabet et al., 2018). C/EBPα,β dKO B cells were retrovirally infected with the LAP*-FKBP12^{F36V} construct, selected for completion of myeloid transdifferentiation by omission of β-mercaptoethanol, and sorted for CD11b$^+$ cells to obtain a myeloid population termed CEBP-dKO-LAP*-FKBP12^{F36V}-GMPBTs. dTAG-13 treatment of the cells removed the LAP*-FKBP12^{F36V} construct (Figure S3C), whereas the monovalent FKBP12^{F36V}-binding compounds AP1867 and FK506 (at 5 μM) slightly enhanced the level of the chimeric LAP*-FKBP12^{F36V} protein, in accordance with their known stabilization effect on FKBP12 chimeras. A 72-h treatment of LAP*-FKBP12^{F36V}-GMPBT with dTAG-13, AP1867, or FK506 revealed alternative progenitor differentiation paths (Figures 3C and 3D). The targeted degradation of LAP*-FKBP12^{F36V} increased the CD115$^+$ population from the initial 18%–95% (Figure 3C) with a concomitant loss of the progenitor population (Ly6G$^-$ CD115$^-$ DN, decreased from 75% to <4%). Concomitantly, dTAG-13 treatment completely abolished the Ly6G$^+$ Neu population (from 3% to <0.5%), whereas stabilizing the LAP*-FKBP12^{F36V} construct with either FK506 or AP1867 increased the Ly6G$^+$ Neu population from the initial 3% to 10% and 12%, respectively (Figure 3D). The increased presence of cells with monocyte/macrophage morphology (enlarged size and extended vacuoles) when treated with dTAG-13 supported the flow cytometry analysis results (Figure S3D). These data demonstrated that LAP* was required for the maintenance of the progenitor state and for Neu differentiation but unnecessary for macrophage differentiation and maintenance.

The v-Abl oncoprotein complements the CSF2 dependency of GMPBTs

Finally, we examined the requirement for the v-Abl kinase after completion of GMPBT transdifferentiation. Pharmacological inhibition of the v-Abl kinase by imatinib compromised GMPBT survival and proliferation already at very low concentrations and completely abrogated cell survival at concentrations greater than 0.4 μM (Figure 4A). In B cells, the v-Abl tyrosine kinase overcame cytokine dependency, which enabled exploration of whether it might likewise replace myelomonotrophic cytokine signals in GMPBTs. Accordingly, GMPBTs were cultured in the presence of imatinib to inhibit v-Abl and supplemented with cytokines or cytokine cocktails to rescue cell survival and proliferation. As shown in Figure 4B, CSF2 (granulocyte-macrophage colony-stimulating factor [GM-CSF]) alone rescued the survival and proliferation of imatinib-treated GMPBTs, while stem cell factor (SCF), interleukin-3 (IL-3), IL-6, CSF1 (macrophage [M]-CSF), or CSF3 (granulocyte [G]-CSF) alone or combined with IL-3 and SCF did not or only marginally prevented cell death. The titration of CSF1, CSF2, and CSF3 in imatinib-treated GMPBTs demonstrated a dose-response relationship for CSF2, with approximately 10 ng/mL CSF2 as the optimal concentration to support survival and proliferation, whereas even high concentrations of either CSF1 or CSF3 failed to rescue v-Abl inhibitor-treated GMPBTs (Figure 4C). Morphological inspection of the cells treated with CSF2 and imatinib revealed that the addition of CSF1 or CSF3 nevertheless selectively supported the final stages of monopoiesis and granulopoiesis, respectively (Figure 4D). These data supported the idea that GMPBTs maintained intact cytokine signaling pathways and recapitulated the differentiation of innate immune cells into macrophages and granulocytes.

CSF2 binds to a hetero-multimeric CSF2 cell-surface receptor and activates the JAK2-STAT5 pathway (Becher et al., 2016). To examine the importance of the JAK2-STAT5 pathway in GMPBTs, we first tested whether a JAK2 kinase inhibitor (ruxolitinib) would also inhibit growth of GMPBTs. We cultured GMPBTs in the presence of imatinib, CSF2, and ruxolitinib (Figure 4E). Ruxolitinib did not affect GMPBTs when the v-Abl kinase remained active. However, ruxolitinib abrogated the rescue by CSF2 in a concentration-dependent manner when the v-Abl kinase was simultaneously inhibited by imatinib (schematically depicted in Figure 4G). These results are in accordance with the observation that the Abl oncoprotein bypassed the requirement for JAK2 activation and abrogated cytokine dependence (Hayashi et al., 2011). Next, we examined whether ectopic expression of the TF STAT5 would also overcome the v-Abl dependency of GMPBTs. The activation of conditional versions of hydroxytamoxifen-inducible WT STAT5a-ER^T or a constitutively active STAT5a-cS5F-ER^T chimera both complemented (50%–70%) v-Abl kinase inactivation (Figure 4F), while activation of the defective STAT5Δ749-ER^T or empty vector did not (Javadi et al., 2013; Moriggl et al., 1996). These data demonstrated that v-Abl signaling short-circuits the CSF2 dependency of GMPBTs and alternatively activates STAT5 (summarized in Figure 4G), similarly to the major function of the human BCR-ABL translocation oncoprotein in chronic-phase CML LSCs (Hantschel et al., 2012; Hoelbl et al., 2010; Moriggl et al., 2005).

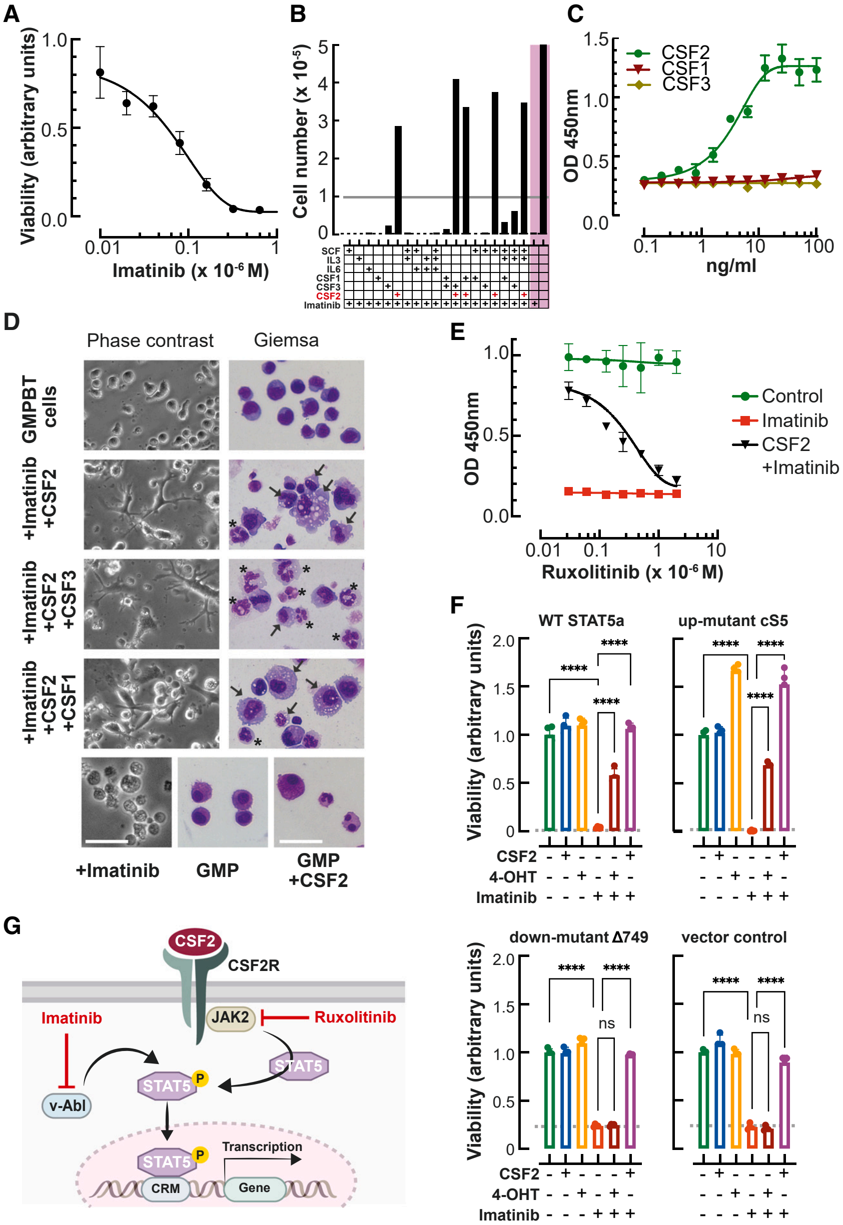

Figure 4. Cytokine signaling and v-Abl dependency of GMPBTs

(A) Cell viability/proliferation as determined by WST-1 colorimetric assay of GMPBTs treated with various concentrations of the v-Abl kinase inhibitor imatinib (n = 3, sigmoidal curve fit, four-parameter logistic, R^2 = 0.97). Viability of cells was determined 48 h post treatment.

(B) Survival of GMPBTs treated with imatinib and supplemented with cytokines as indicated. Cells were seeded at 1 × 10^5 cells (indicated by a gray line), and viable cells were counted after 48 h (toluidine blue exclusion, n = 4, data are shown as mean of independent cell counts from microscopic inspection). Control groups without cytokine supplementation are highlighted on a magenta background on the right.

(C) Titration of CSF1 (M-CSF, red), CSF2 (GM-CSF, green), and CSF3 (G-CSF, brown) in the presence of imatinib (0.6 μM). Cell proliferation and viability were measured after 48 h as determined by WST-1 colorimetric assay (n = 3, sigmoidal curve fit, four-parameter logistic, R^2 = 0.93). p values of data interpolation: CSF1, p = 0.056; CSF2, p = 0.0001; CSF3, p = 0.022.

(D) Morphology of GMPBTs with or without imatinib and cytokine treatment, as indicated on the left. Phase-contrast (cell culture samples) and May-Grünwald-Giemsa staining (cytospins) on day 3 post treatment. Arrows indicate cells with macrophage morphology (enlarged and extended cell body and vacuoles), and asterisks indicate cells with Neu morphology (ring-shaped or lobular nuclei, azurophilic cytoplasmic granules). Controls are shown in the bottom row, including GMPBTs treated with imatinib (apoptotic cells, left), stained cytospins of GMP cells isolated from WT mouse bone marrow ($Lin^-cKit^+Sca\text{-}1^-Fcgr3^+Ly\text{-}6C^-$, center), and GMP cells treated with CSF2 for 2 days in culture (right). Scale bar, 50 μm.

(E) Ruxolitinib sensitivity of GMPBTs. GMPBTs were treated with ruxolitinib (an inhibitor of Jak2) in the presence or absence of imatinib (an inhibitor of v-Abl). Ruxolitinib sensitivity emerged only in the presence of imatinib and CSF2 (CSF2, 10 ng/mL; imatinib, 0.6 μM, black line) but not in the absence of imatinib (green line), indicating that Jak2 and v-Abl are functionally redundant in GMPBTs. Viability of cells was determined 48 h post treatment using a WST-1 colorimetric cell viability assay (n = 4, sigmoidal curve fit, four-parameter logistic, R^2 = 0.96).

(F) Viability of imatinib treated GMPBTs supplemented with various retrovirally delivered conditional STAT5 TF constructs. Conditional 4-hydroxytamoxifen (4-OHT)-dependent activation of WT STAT5, a constitutively active STAT5 mutant (cS5), an inactive STAT5 mutant (D749), or vector as a negative control is shown at the top of each bar graph. CSF2-imatinib-treated GMPBTs served as a positive control. Viability of cells was determined 48 h post treatment using a WST-1 colorimetric cell viability assay. Dashed lines indicate viability of cells prior to treatment.

Data are mean ± SD from four independent experiments; one-way ANOVA with Turkey's multiple comparisons tests, ****$p < 0.0001$; ns, not significant.

(G) Schematic representation of the CSF2-JAK2-STAT5 signaling. CRM, cis-regulatory module.

DISCUSSION

We demonstrated that C/EBPβ-LAP* transdifferentiates v-Abl-transformed pre-B cells in tissue culture to adopt a sustainable GMP-like phenotype, called GMPBTs. v-Abl or C/EBPβ-LAP* did not hinder continuous GM-lineage specification and differentiation but, rather, facilitated autonomous progenitor self-renewal and proliferation. GMPBTs thus resemble self-renewing GMPs or LSCs that can be easily expanded in tissue culture and retain intrinsic granulocyte and macrophage differentiation capacity.

The GMPBT model represents a surrogate GMP/LSC proxy that can be easily scaled up and manipulated experimentally. It allows exploitation of already existing genetically modified mouse models to explore myeloid commitment and differentiation processes. The GMPBT system holds promise for elucidating the molecular and biochemical mechanisms of myeloid cell diversification and uncovering the genetic and proteomic factors that underlie the respective phenotypes. Furthermore, the GMPBT system can help to reduce and replace animal experiments, aligning with the important principles of the 3R framework: reduce, replace, refine (Festing and Wilkinson, 2007). The GMPBT system is positioned as a versatile and accessible tool that enables scientists from non-hematological disciplines to conduct experiments that contribute to advancing our understanding of cell lineage specification and lineage plasticity.

A GMP-like *in vitro* model

The GMPBT population exhibits a continuous release of cells undergoing spontaneous differentiation, while a fraction of the population maintains a self-renewing progenitor state. In contrast to regular GMP cells, individual cell transcriptomes revealed that the GMPBT population contained an enlarged portion of immature and undetermined cells with myelomonocytic multilineage potential. The amount of such immature cellular stages is uncommon in mouse bone marrow progenitors but become more prevalent after tissue culture propagation of HSCs or genetic interference with master TFs or in the pre-leukemic state (Ceredig et al., 2009; Paul et al., 2015; Weinreb et al., 2020). The heterogeneous GMPBT population contains progenitor subsets that express non-overlapping early markers such as *Vcam1* (Figure S2K), *Spn*, or *Clec12a* and may help to explore initiating mechanisms involved in neighboring lineage choices.

An alternative experimental strategy for investigating mouse GMP biology involves ectopic expression of conditional versions of HoxB8 or HoxA9 in $Lin^{-}Kit^{+}$ progenitors. These pseudo-transformed progenitors can sustain cytokine-dependent *in vitro* propagation and can be driven toward differentiation; e.g., into granulocytes or macrophages by inactivation of the Hox component and with the aid of specific cytokine supplements (Sykes et al., 2016; Wang et al., 2006). In contrast, the constitutively active v-Abl kinase oncoprotein in combination with C/EBPβ-LAP* enables cytokine-independent *in vitro* expansion, resembling GMPs/LSCs. The potential to pharmacologically inhibit the v-Abl kinase in conjunction with conditional and interactome-constrained C/EBP-β-LAP* mutant constructs furnishes an experimental toolkit for unveiling latent molecular mechanisms governing transdifferentiation, cell fate determination, and trajectories of differentiation (Dittmar et al., 2019; Stoilova et al., 2013).

TFs and signaling pathways involved in GMPBT diversification

The orchestration of hematopoietic lineage hierarchy depends on the interplay between cross-regulatory TFs and upstream signaling networks. These TFs serve as the linchpins that establish distinctive gene expression patterns, driving the diversification of hematopoiesis. In addition to cell differentiation, the pivotal GMP TFs (namely, C/EBPs, PU.1, and STAT5) also play an integral role in triggering myelogenous leukemia (Pucella et al., 2020). In addition, the intricate control of lineage allocation in granulopoietic and monopoietic pathways is connected to TFs such as Gfi1, IRF8, and KLF4 that are a part of the CoRC to determine the destinies of sibling cells (Arendt et al., 2016).

Irf8 and *Cebpb* exhibit interconnected auto-regulatory loops that exert cross-inhibitory effects on each other, thereby playing an important role in determining alternate cell destinies (Bornstein et al., 2014; Graf and Enver, 2009). In murine models, the elimination of *Irf8* strongly curtailed the production of monocytes while concurrently promoting granulopoiesis, an outcome that was recapitulated in GMPBTs after targeted removal of the *Irf8* gene (Holtschke et al., 1996; Tamura et al., 2015; Yanez et al., 2015). These data solidify the notion that GMPBTs emulate the lineage commitment features observed in regular GMPs. While removal of IRF8 did not diminish the progenitor fraction, targeted proteolysis of C/EBPβ revealed its importance for maintaining the progenitor state in accordance with the finding that C/EBPβ can effectively replace C/EBPα in establishing and sustaining the GMP state. The observation that elimination of ectopically expressed C/EBPβ in dKO failed to revert the lymphoid phenotype supports the existence of a CEBP-independent mechanism for retaining epigenetic memory subsequent to the acquisition of the monocyte fate.

GMPBT biology suggests a key role of C/EBPβ in transformation

The reliance of GMPBT proliferation on C/EBPβ resembles the previously established C/EBP dependency in MLL-transformed myelomonocytic progenitors (Wesolowski et al., 2021). How does C/EBPβ support myelogenous transformation by Abl kinase oncogenes? In CML, the BCR-ABL fusion protein short-circuits CSF2 signaling via downstream activation of STAT5 (Carlesso et al., 1996; Hantschel et al., 2012; Minami et al., 2008). Notably, phosphorylation of a critical tyrosine residue within the transactivation domain of C/EBPβ by c-Abl or related Arg kinases has been demonstrated to stabilize C/EBPβ. This phosphorylation event prevents the interaction between C/EBPβ and the pseudokinases Tribble 1 and 2, which mediate C/EBPβ degradation via the COP1-Cul4-proteasome pathway (Li et al., 2009; Ndoja et al., 2020). Building on these published data, we hypothesized that v-Abl fosters C/EBPβ stability to synergize with activated STAT5, thereby preserving the progenitor state. Analogously, emergency granulopoiesis hinges on C/EBPβ and requires upstream signals culminating in STAT5 activation (Hirai et al., 2006; Kimura et al., 2009). Therefore, GMPBTs appear akin to progenitors in a GMP/LSC state that are undergoing emergency granulopoiesis (Hayashi et al., 2011; Herault et al., 2017; Hirai et al., 2015; Jamieson et al., 2004). As a result, GMPBTs offer a promising avenue for comprehensive exploration of emergency granulopoiesis and the phenomenon of "non-oncogenic addiction" to C/EBPβ (Nagel et al., 2016). This dependency on C/EBPβ might have implications beyond myelogenous leukemia. Enhanced C/EBPβ expression has been correlated with several other cancers, such as breast cancer and multiple myeloma (Ewen and Lamb, 2004; Musgrove et al., 2011). Consequently, investigating the potential pharmacological inhibition of C/EBPβ holds promise as a strategy to unveil potential vulnerability for therapeutic intervention in cancer.

In summary, we represent here an experimental model of C/EBPβ-dependent B cell transdifferentiation, which leads to the generation of an immature GMP-like population with the potential to differentiate into monocytes/macrophages and Neus. Our *in vitro*-generated GMP-like cells show similarities to their *ex vivo* counterparts, and genetic manipulations further confirm the importance of IRF8, STAT5, and C/EBPβ in successful transdifferentiation. This system has the potential to replace costly, inefficient, and invasive *in vivo* methods for studying B cell transdifferentiation.

EXPERIMENTAL PROCEDURES

For further details, see supplemental experimental procedures.

Resource availability

Corresponding authors

Achim Leutz: aleutz@mdc-berlin.de.

Materials availability

Requests for additional raw and analyzed data as well as materials will be promptly received and reviewed by the corresponding author to verify whether the request is subject to any intellectual property of confidentiality obligations. Any data and materials that can be shared will be released via a material transfer agreement.

Data and code availability

The scRNA-seq data are publicly available at the National Center for Biotechnology Information (GEO: GSE248415).

Cells and transdifferentiation

v-Abl-transformed *Cebpa*$^{fl/fl}$*Cebpb*$^{fl/fl}$ pre-B cells (WT B cells) were generated from pre/pro-B cells from C57BL/6J mice and cultured as described previously (Cirovic et al., 2017). Briefly, B cells were retrovirally infected with the C/EBPβ-LAP* construct by spinoculation at 37°C and 2,000 rpm for 60 min before overnight cultivation. Medium was exchanged 24 h later. Transdifferentiated cells emerged as early as 1 day p.i. and manifested at days 4–6 p.i. Further details regarding genetic engineering and selection are described in the Supplemental experimental procedures.

Droplet-based scRNA-seq

C/EBPβ-LAP*-induced transdifferentiation was performed on the WT B cells and dKO B cells in parallel. On day 6 p.i., 5,000 EGFP^{+} cells were sorted into phosphate-buffered saline (PBS) and processed at the Single Cell Technologies Unit (Scientific Genomics Platforms, Max Delbrück Center for Molecular Medicine, Berlin, Germany) following the standard 10X Genomics workflow. Briefly, the cells were partitioned and barcoded using Chromium Automation (10X Genomics), followed by standard library preparation, quality control, and sequencing. Chromium Single Cell 3′ v2 chemistry was used for both cell types. Sequencing was performed with a HiSeq400 unit (Illumina) with an 8-bp index read. For WT B cell transdifferentiation, 3,297 cells were processed with an average sequencing saturation of 70.3%. For dKO B cell transdifferentiation, 1,423 cells were processed with an average sequencing saturation of 89%. See supplemental experimental procedures for a detailed bioinformatics analysis.

Flow cytometry analysis and sorting

For flow cytometry analysis and fluorescence-activated cell sorting (FACS), the cells were harvested into 5-mL tubes and washed with FACS buffer (2% fetal bovine serum, 2 mM EDTA in PBS). The cells were incubated in Fc block solution (TruStain FcX, anti-mouse CD16/32, BioLegend) at 4°C for 10 min and then stained with a cocktail of fluorophore-conjugated anti-mouse antibodies against CD19, CD11b, Ly6G, and CD115 at 4°C in the dark for 30 min. The stained cells were washed twice and resuspended in FACS buffer containing propidium iodide (PI, BD Biosciences) for live/dead cell discrimination. For each experiment, unstained cells (B cells and/or GMPBTs), single staining and fluorescence-minus-one staining samples were used as controls. Marker expression

was measured on an LSRFortessa unit (BD Biosciences), and cells were sorted using FACSAria II/III units (BD Biosciences). For scRNA-seq experiments, the cells were sorted directly into ice-cold PBS containing 0.04% bovine serum albumin and processed.

Cell viability and growth

For the colorimetric WST-1 assay, 3×10^4 cells were suspended in 100 μL medium in flat-bottomed microplates. WST-1 reagent (10 μL, Roche) was added at the indicated times, and the microplates were incubated under cell culture conditions. The absorbance at 450-nm wavelength was determined after 30 and 60 min against the blank control absorbance (cell culture medium without cells). For manual cell counting, the cell suspension was mixed with trypan blue (Sigma-Aldrich), transferred to a Neubauer hemocytometer, and counted in quadruplicate.

SUPPLEMENTAL INFORMATION

Supplemental information can be found online at https://doi.org/10.1016/j.stemcr.2023.11.011.

ACKNOWLEDGMENTS

We thank Hans-Peter Rahn and Kirstin Rautenberg (MDC, Berlin, Germany) for flow cytometry support; Cornelius Fischer and Caroline Braeuning (MDC-BIH, Berlin, Germany) for 10X Genomics Chromium scRNA-seq support; Henrik E. Mei, Axel Schulz, and Heike Hirseland (DRFZ, Berlin, Germany) for LEGENDScreen support; and Richard Moriggl (University of Veterinary Medicine, Vienna, Austria) for discussions and reagents.

AUTHOR CONTRIBUTIONS

L.T.N., investigation, original draft, visualization, and validation; K.Z., investigation, formal analysis, data curation, and validation; E.K.-L., methodology, resources and validation; R.L., investigation and validation; M.H., resources and investigation; A.M., data curation, review & editing, and supervision; A.L., conceptualization, supervision, project administration, funding acquisition, methodology, investigation, and review & editing.

DECLARATION OF INTERESTS

The authors declare no competing interests.

Received: December 22, 2022
Revised: November 27, 2023
Accepted: November 28, 2023
Published date: December 28, 2023

REFERENCES

Arendt, D., Musser, J.M., Baker, C.V.H., Bergman, A., Cepko, C., Erwin, D.H., Pavlicev, M., Schlosser, G., Widder, S., Laubichler, M.D., and Wagner, G.P. (2016). The origin and evolution of cell types. Nat. Rev. Genet. *17*, 744–757.

Becher, B., Tugues, S., and Greter, M. (2016). GM-CSF: From Growth Factor to Central Mediator of Tissue Inflammation. Immunity *45*, 963–973.

Bornstein, C., Winter, D., Barnett-Itzhaki, Z., David, E., Kadri, S., Garber, M., and Amit, I. (2014). A negative feedback loop of transcription factors specifies alternative dendritic cell chromatin States. Mol. Cell *56*, 749–762.

Bussmann, L.H., Schubert, A., Vu Manh, T.P., De Andres, L., Desbordes, S.C., Parra, M., Zimmermann, T., Rapino, F., Rodriguez-Ubreva, J., Ballestar, E., and Graf, T. (2009). A robust and highly efficient immune cell reprogramming system. Cell Stem Cell *5*, 554–566.

Carlesso, N., Frank, D.A., and Griffin, J.D. (1996). Tyrosyl phosphorylation and DNA binding activity of signal transducers and activators of transcription (STAT) proteins in hematopoietic cell lines transformed by Bcr/Abl. J. Exp. Med. *183*, 811–820.

Ceredig, R., Rolink, A.G., and Brown, G. (2009). Models of haematopoiesis: seeing the wood for the trees. Nat. Rev. Immunol. *9*, 293–300.

Cirovic, B., Schönheit, J., Kowenz-Leutz, E., Ivanovska, J., Klement, C., Pronina, N., Bégay, V., and Leutz, A. (2017). C/EBP-Induced Transdifferentiation Reveals Granulocyte-Macrophage Precursor-like Plasticity of B Cells. Stem Cell Rep. *8*, 346–359.

Dittmar, G., Hernandez, D.P., Kowenz-Leutz, E., Kirchner, M., Kahlert, G., Wesolowski, R., Baum, K., Knoblich, M., Hofstätter, M., Muller, A., et al. (2019). PRISMA: Protein Interaction Screen on Peptide Matrix Reveals Interaction Footprints and Modifications-Dependent Interactome of Intrinsically Disordered C/EBPbeta. iScience *13*, 351–370.

Dress, R.J., Liu, Z., and Ginhoux, F. (2020). Towards the Better Understanding of Myelopoiesis Using Single-Cell Technologies. Mol. Immunol. *122*, 186–192.

Evrard, M., Kwok, I.W.H., Chong, S.Z., Teng, K.W.W., Becht, E., Chen, J., Sieow, J.L., Penny, H.L., Ching, G.C., Devi, S., et al. (2018). Developmental Analysis of Bone Marrow Neutrophils Reveals Populations Specialized in Expansion, Trafficking, and Effector Functions. Immunity *48*, 364–379.e8.

Ewen, M.E., and Lamb, J. (2004). The activities of cyclin D1 that drive tumorigenesis. Trends Mol. Med. *10*, 158–162.

Feng, J., Wang, H., Shin, D.M., Masiuk, M., Qi, C.F., and Morse, H.C., 3rd. (2011). IFN regulatory factor 8 restricts the size of the marginal zone and follicular B cell pools. J. Immunol. *186*, 1458–1466.

Festing, S., and Wilkinson, R. (2007). The ethics of animal research. Talking Point on the use of animals in scientific research. EMBO Rep. *8*, 526–530.

Giladi, A., Paul, F., Herzog, Y., Lubling, Y., Weiner, A., Yofe, I., Jaitin, D., Cabezas-Wallscheid, N., Dress, R., Ginhoux, F., et al. (2018). Single-cell characterization of haematopoietic progenitors and their trajectories in homeostasis and perturbed haematopoiesis. Nat. Cell Biol. *20*, 836–846.

Graf, T., and Enver, T. (2009). Forcing cells to change lineages. Nature *462*, 587–594.

Hantschel, O., Warsch, W., Eckelhart, E., Kaupe, I., Grebien, F., Wagner, K.U., Superti-Furga, G., and Sexl, V. (2012). BCR-ABL uncouples canonical JAK2-STAT5 signaling in chronic myeloid leukemia. Nat. Chem. Biol. *8*, 285–293.

Hayashi, Y., Hirai, H., Yao, H., Yoshioka, S., Satake, S., Kamio, N., Miura, Y., Ashihara, E., Fujiyama, Y., Tenen, D.G., and Maekawa, T. (2011). BCR/ABL-Mediated Myeloid Expansion Is Promoted by C/EBPβ, a Regulator of Emergency Granulopoiesis. Blood *118*, 3747.

Heng, T.S.P., and Painter, M.W.; Immunological Genome Project Consortium (2008). The Immunological Genome Project: networks of gene expression in immune cells. Nat. Immunol. *9*, 1091–1094.

Hérault, A., Binnewies, M., Leong, S., Calero-Nieto, F.J., Zhang, S.Y., Kang, Y.A., Wang, X., Pietras, E.M., Chu, S.H., Barry-Holson, K., et al. (2017). Myeloid progenitor cluster formation drives emergency and leukaemic myelopoiesis. Nature *544*, 53–58.

Hirai, H., Yokota, A., Tamura, A., Sato, A., and Maekawa, T. (2015). Non-steady-state hematopoiesis regulated by the C/EBPbeta transcription factor. Cancer Sci. *106*, 797–802.

Hirai, H., Zhang, P., Dayaram, T., Hetherington, C.J., Mizuno, S.i., Imanishi, J., Akashi, K., and Tenen, D.G. (2006). C/EBPbeta is required for 'emergency' granulopoiesis. Nat. Immunol. *7*, 732–739.

Hoelbl, A., Schuster, C., Kovacic, B., Zhu, B., Wickre, M., Hoelzl, M.A., Fajmann, S., Grebien, F., Warsch, W., Stengl, G., et al. (2010). Stat5 is indispensable for the maintenance of bcr/abl-positive leukaemia. EMBO Mol. Med. *2*, 98–110.

Holtschke, T., Löhler, J., Kanno, Y., Fehr, T., Giese, N., Rosenbauer, F., Lou, J., Knobeloch, K.P., Gabriele, L., Waring, J.F., et al. (1996). Immunodeficiency and chronic myelogenous leukemia-like syndrome in mice with a targeted mutation of the ICSBP gene. Cell *87*, 307–317.

Ito, Y., Nakahara, F., Kagoya, Y., and Kurokawa, M. (2021). CD62L expression level determines the cell fate of myeloid progenitors. Stem Cell Rep. *16*, 2871–2886.

Iwasaki, H., and Akashi, K. (2007). Myeloid Lineage Commitment from the Hematopoietic Stem Cell. Immunity *26*, 726–740.

Jamieson, C.H.M., Ailles, L.E., Dylla, S.J., Muijtjens, M., Jones, C., Zehnder, J.L., Gotlib, J., Li, K., Manz, M.G., Keating, A., et al. (2004). Granulocyte-macrophage progenitors as candidate leukemic stem cells in blast-crisis CML. N. Engl. J. Med. *351*, 657–667.

Javadi, M., Richmond, T.D., Huang, K., and Barber, D.L. (2013). CBL linker region and RING finger mutations lead to enhanced granulocyte-macrophage colony-stimulating factor (GM-CSF) signaling via elevated levels of JAK2 and LYN. J. Biol. Chem. *288*, 19459–19470.

Kimura, A., Rieger, M.A., Simone, J.M., Chen, W., Wickre, M.C., Zhu, B.M., Hoppe, P.S., O'Shea, J.J., Schroeder, T., and Hennighausen, L. (2009). The transcription factors STAT5A/B regulate GM-CSF-mediated granulopoiesis. Blood *114*, 4721–4728.

Krivtsov, A.V., Twomey, D., Feng, Z., Stubbs, M.C., Wang, Y., Faber, J., Levine, J.E., Wang, J., Hahn, W.C., Gilliland, D.G., et al. (2006). Transformation from committed progenitor to leukaemia stem cell initiated by MLL-AF9. Nature *442*, 818–822.

Kurotaki, D., Nakabayashi, J., Nishiyama, A., Sasaki, H., Kawase, W., Kaneko, N., Ochiai, K., Igarashi, K., Ozato, K., Suzuki, Y., and Tamura, T. (2018). Transcription Factor IRF8 Governs Enhancer Landscape Dynamics in Mononuclear Phagocyte Progenitors. Cell Rep. *22*, 2628–2641.

Kurotaki, D., Yamamoto, M., Nishiyama, A., Uno, K., Ban, T., Ichino, M., Sasaki, H., Matsunaga, S., Yoshinari, M., Ryo, A., et al. (2014). IRF8 inhibits C/EBPalpha activity to restrain mononuclear phagocyte progenitors from differentiating into neutrophils. Nat. Commun. *5*, 4978.

Kwok, I., Becht, E., Xia, Y., Ng, M., Teh, Y.C., Tan, L., Evrard, M., Li, J.L.Y., Tran, H.T.N., Tan, Y., et al. (2020). Combinatorial Single-Cell Analyses of Granulocyte-Monocyte Progenitor Heterogeneity Reveals an Early Uni-potent Neutrophil Progenitor. Immunity *53*, 303–318.e5.

Li, X., Liu, X., Wang, G., Zhu, X., Qu, X., Li, X., Yang, Y., Peng, L., Li, C., Li, P., et al. (2009). Non-receptor tyrosine kinases c-Abl and Arg regulate the activity of C/EBPbeta. J. Mol. Biol. *391*, 729–743.

Liu, Z., Gu, Y., Chakarov, S., Bleriot, C., Kwok, I., Chen, X., Shin, A., Huang, W., Dress, R.J., Dutertre, C.A., et al. (2019). Fate Mapping via Ms4a3-Expression History Traces Monocyte-Derived Cells. Cell *178*, 1509–1525.e19.

Mildner, A., Schonheit, J., Giladi, A., David, E., Lara-Astiaso, D., Lorenzo-Vivas, E., Paul, F., Chappell-Maor, L., Priller, J., Leutz, A., et al. (2017). Genomic Characterization of Murine Monocytes Reveals C/EBPbeta Transcription Factor Dependence of Ly6C(-) Cells. Immunity *46*, 849–862.e7.

Minami, Y., Stuart, S.A., Ikawa, T., Jiang, Y., Banno, A., Hunton, I.C., Young, D.J., Naoe, T., Murre, C., Jamieson, C.H.M., and Wang, J.Y.J. (2008). BCR-ABL-transformed GMP as myeloid leukemic stem cells. Proc. Natl. Acad. Sci. USA *105*, 17967–17972.

Moriggl, R., Gouilleux-Gruart, V., Jähne, R., Berchtold, S., Gartmann, C., Liu, X., Hennighausen, L., Sotiropoulos, A., Groner, B., and Gouilleux, F. (1996). Deletion of the carboxyl-terminal transactivation domain of MGF-Stat5 results in sustained DNA binding and a dominant negative phenotype. Mol. Cell Biol. *16*, 5691–5700.

Moriggl, R., Sexl, V., Kenner, L., Duntsch, C., Stangl, K., Gingras, S., Hoffmeyer, A., Bauer, A., Piekorz, R., Wang, D., et al. (2005). Stat5 tetramer formation is associated with leukemogenesis. Cancer Cell *7*, 87–99.

Musgrove, E.A., Caldon, C.E., Barraclough, J., Stone, A., and Sutherland, R.L. (2011). Cyclin D as a therapeutic target in cancer. Nat. Rev. Cancer *11*, 558–572.

Nabet, B., Roberts, J.M., Buckley, D.L., Paulk, J., Dastjerdi, S., Yang, A., Leggett, A.L., Erb, M.A., Lawlor, M.A., Souza, A., et al. (2018). The dTAG system for immediate and target-specific protein degradation. Nat. Chem. Biol. *14*, 431–441.

Nagel, R., Semenova, E.A., and Berns, A. (2016). Drugging the addict: non-oncogene addiction as a target for cancer therapy. EMBO Rep. *17*, 1516–1531.

Naik, S.H., Perié, L., Swart, E., Gerlach, C., van Rooij, N., de Boer, R.J., and Schumacher, T.N. (2013). Diverse and heritable lineage imprinting of early haematopoietic progenitors. Nature *496*, 229–232.

Ndoja, A., Reja, R., Lee, S.H., Webster, J.D., Ngu, H., Rose, C.M., Kirkpatrick, D.S., Modrusan, Z., Chen, Y.J.J., Dugger, D.L., et al.

(2020). Ubiquitin Ligase COP1 Suppresses Neuroinflammation by Degrading c/EBPbeta in Microglia. Cell *182*, 1156–1169.e12.

Niederkorn, M., and Starczynowski, D.T. (2017). GMP-ing to Spatial Conclusions about Emergency and Leukemic Myelopoiesis. Cell Stem Cell *20*, 579–581.

Paul, F., Arkin, Y., Giladi, A., Jaitin, D.A., Kenigsberg, E., Keren-Shaul, H., Winter, D., Lara-Astiaso, D., Gury, M., Weiner, A., et al. (2015). Transcriptional Heterogeneity and Lineage Commitment in Myeloid Progenitors. Cell *163*, 1663–1677.

Pucella, J.N., Upadhaya, S., and Reizis, B. (2020). The Source and Dynamics of Adult Hematopoiesis: Insights from Lineage Tracing. Annu. Rev. Cell Dev. Biol. *36*, 529–550.

Qin, Q., Fan, J., Zheng, R., Wan, C., Mei, S., Wu, Q., Sun, H., Brown, M., Zhang, J., Meyer, C.A., and Liu, X.S. (2020). Lisa: inferring transcriptional regulators through integrative modeling of public chromatin accessibility and ChIP-seq data. Genome Biol. *21*, 32.

Regalo, G., and Leutz, A. (2013). Hacking cell differentiation: transcriptional rerouting in reprogramming, lineage infidelity and metaplasia. EMBO Mol. Med. *5*, 1154–1164.

Reimand, J., Arak, T., Adler, P., Kolberg, L., Reisberg, S., Peterson, H., and Vilo, J. (2016). g:Profiler-a web server for functional interpretation of gene lists (2016 update). Nucleic Acids Res. *44*, W83–W89.

Stoilova, B., Kowenz-Leutz, E., Scheller, M., and Leutz, A. (2013). Lymphoid to myeloid cell trans-differentiation is determined by C/EBPbeta structure and post-translational modifications. PLoS One *8*, e65169.

Street, K., Risso, D., Fletcher, R.B., Das, D., Ngai, J., Yosef, N., Purdom, E., and Dudoit, S. (2018). Slingshot: cell lineage and pseudotime inference for single-cell transcriptomics. BMC Genom. *19*, 477.

Sykes, D.B., Kfoury, Y.S., Mercier, F.E., Wawer, M.J., Law, J.M., Haynes, M.K., Lewis, T.A., Schajnovitz, A., Jain, E., Lee, D., et al. (2016). Inhibition of Dihydroorotate Dehydrogenase Overcomes Differentiation Blockade in Acute Myeloid Leukemia. Cell *167*, 171–186.e15.

Tamura, T., Kurotaki, D., and Koizumi, S.i. (2015). Regulation of Myelopoiesis by the Transcription Factor IRF8. International Journal of Hematology (Springer-Verlag Tokyo).

Wang, G.G., Calvo, K.R., Pasillas, M.P., Sykes, D.B., Häcker, H., and Kamps, M.P. (2006). Quantitative production of macrophages or neutrophils ex vivo using conditional Hoxb8. Nat. Methods *3*, 287–293.

Weinreb, C., Rodriguez-Fraticelli, A., Camargo, F.D., and Klein, A.M. (2020). Lineage tracing on transcriptional landscapes links state to fate during differentiation. Science *367*, eaaw3381.

Wesolowski, R., Kowenz-Leutz, E., Zimmermann, K., Dörr, D., Hofstätter, M., Slany, R.K., Mildner, A., and Leutz, A. (2021). Myeloid transformation by MLL-ENL depends strictly on C/EBP. Life Sci. Alliance *4*, e202000709–e202000714.

Xie, H., Ye, M., Feng, R., and Graf, T. (2004). Stepwise reprogramming of B cells into macrophages. Cell *117*, 663–676.

Yanez, A., Coetzee, S.G., Olsson, A., Muench, D.E., Berman, B.P., Hazelett, D.J., Salomonis, N., Grimes, H.L., and Goodridge, H.S. (2017). Granulocyte-Monocyte Progenitors and Monocyte-Dendritic Cell Progenitors Independently Produce Functionally Distinct Monocytes. Immunity *47*, 890–902.e4.

Yáñez, A., Ng, M.Y., Hassanzadeh-Kiabi, N., and Goodridge, H.S. (2015). IRF8 acts in lineage-committed rather than oligopotent progenitors to control neutrophil vs monocyte production. Blood *125*, 1452–1459.

Ye, M., Zhang, H., Yang, H., Koche, R., Staber, P.B., Cusan, M., Levantini, E., Welner, R.S., Bach, C.S., Zhang, J., et al. (2015). Hematopoietic Differentiation Is Required for Initiation of Acute Myeloid Leukemia. Cell Stem Cell *17*, 611–623.

Stem Cell Reports, Volume *19*

Supplemental Information

C/EBPβ-induced lymphoid-to-myeloid transdifferentiation emulates granulocyte-monocyte progenitor biology

Linh Thuy Nguyen, Karin Zimmermann, Elisabeth Kowenz-Leutz, Ramonique Lim, Maria Hofstätter, Alexander Mildner, and Achim Leutz

Legends to Supplementary Figures, Nguyen et al.

Supplementary Figure S1 (related to Figure 1)

A. Flow cytometric analysis of *Cebpa*$^{fl/fl}$ *Cebpb*$^{fl/fl}$ B cells (WT-B cells) or *Cebpa*$^{-/-}$ *Cebpb*$^{-/-}$ cells (dKO-B cells) transdifferentiation at day 6 p.i. Cells were stained with lineage markers CD19 (B cells), CD11b (myeloid cells), Ly6G (granulocytes) and CD115 (monocytes/macrophages). For scRNA-seq, GFP^{+} populations were sorted as indicated.
B. Expression of top5 differentially expressed marker genes in GMPBT cells. Common genes of Clusters 7 and 8 (*Vpreb1/2/3*, *Ebf1* and *Cd79a*) indicate them as lagging and myeloid primed B cells, respectively.
C. UMAP and clustering of GMPBT derived from dKO- (left, N=1432 cells) and WT-B cells (right, N=3297 cells). All clusters are represented in both samples.
D. Proportion of cells in each cluster comparing WT- and dKO-GMPBT cells. ScRNA-seq data from both WT- and dKO-GMPBT cell pools were integrated and the percentages of cells in each cluster are shown.
E. Expression of the endogenous C/EBP family genes (indicated on top, data derived from scRNA-seq) in myeloid clusters 1-6 are shown for WT- and dKO-GMPBT cells. ****P < 0.0001, ***P < 0.001, **P < 0.01, *P < 0.05, insignificance is not indicated.

Supplementary Figure S2 (related to Figure 2)

A. Gene Set Variation Analysis (GSVA) was applied to compare GMPBT profiling data to published signatures of mouse bone marrow myeloid cells as defined by Giladi et al. (Giladi *et al.*, 2018). Neutrophil signature was used to compute GSVA scores for cells in the GMPBT scRNA-seq data set. Cells with highest scores are indicated in red.
B. Similar to A, monocyte signature from Giladi et al. was used for GSVA.
C. Expression of *Vcam1*, marking pro-neutrophils, across the myeloid Clusters 1-6.

D-G. Gene expression analysis of neutrophil Clusters 1, 2, 3. Genes characteristic for neutrophil progenitors were extracted from Evrard et al. (Evrard *et al.*, 2018), including myeloid transcription factors (D), genes involved in granule production (E), chemotaxis (F, from GO:1903409) and phagocytosis (G, from GO: 0006909).

H. Monocytic gene expression pattern of Clusters 4, 5, 6. Genes identifying monocytes were derived from Mildner et al. (Mildner et al., 2017).

I. GSVA using the signature of DC-like monocytes or Neu-like monocytes, as identified in Weinreb et al. (Weinreb *et al.*, 2020), on monocyte Clusters 4-6. Note that similarities between DC-like monocytes were enriched in Cluster 6, while the Neu-like monocyte signature could be detected preferentially in Cluster 5.

J. Surface marker analysis of GMPBT cells. GMPBT cells transdifferentiated from WT-B cells were subjected to the cell surface marker screening (LegendScreen^{TM}, BioLegend) and processed as described in Materials and Methods. Expressing markers in two independent experiments overlapped, as shown in the Venn's diagram (left). Heatmap (right) shows results of method-optimized experiment 2. The results are presented as percentage of marker-positive cells in each of the GMPBT subsets: Ly6G^{+}, CD115^{+} and double negative (DN).

K. Heatmap representing scRNA-seq expression of genes shortlisted in the heatmap shown in Supplementary Figure S2J. Genes expressed highly in cluster 1 are indicated between the red lines, including *Vcam1*.

L. CD106 (encoded by *Vcam1*) expression in GMPBT subsets as identified in the LegendScreen experiments.

M. Growth curves of isolated CD106 subsets. Two sub-populations of GMPBT cells were sorted: Ly6G^{-}CD115^{-}CD106^{+} (labeled as CD106^{+}) and Ly6G^{-}CD115^{-}CD106^{-} (labeled as CD106^{-}). Sorted cells were seeded at 10^5 cells/mL and cell numbers (N=3) were determined at indicated time points.

N. Differentiation of CD106 subsets. Sorted cells, as shown in Supplementary Figure S2M, were cultivated for 4 days and subjected to flow cytometry analysis for Ly6G and CD115 expression. N=3, P values of Multiple Mann-Whitney t-tests: Ly6G P=0.0286, CD115 P=0.1143.

Supplementary Figure S3 (related to Figure 4)

A. Expression of *Irf8* in scRNA-seq data of WT-GMPBT cells (as in Figure 1A). Note that *Irf8* is expressed in Cluster 4 that relates to the transition/bifurcation of neutrophils and monocyte/macrophages.

B. Representative *Irf8* genotype of Cre-incubated v-Abl transformed *Irf8*$^{fl/fl}$ B cells (PCR analysis). Untreated (-) or Cre-treated cell pool (+Cre) and genotyping of C57BL/6J wild-type mouse (WT) served as controls. Bi-allelic *Irf8*-/- clones (clone 1,2) were subjected to transdifferentiation together with corresponding isogenic control (untreated) clones.

C. Immunoblot analysis of C/EBPβ expression in total protein lysates from C/EBPα,β dKO-B cells transdifferentiated with HA-tagged LAP*-FKBP12^{F36V} (CEBP-dKO-LAP*-FKBP12^{F36V}-GMPBT cells). Cells were treated with 5 µM AP1867, 5 µM FK506, 0.01 µM rapamycin or 5 µM dTAG-13 for 6 hours or 24 hours. Mono-functional AP1867 and FK506 bind to and stabilize FKBP12 while the hetero-bifunctional dTAG-13 selectively degrades the LAP*-FKBP12^{F36V} chimera. Expression of the fusion protein LAP*-FKBP12^{F36V} was detected by an antibody directed against the HA-tag (approx. 55 kDa). Long exposure (top) to demonstrate removal of LAP*-FKBP12^{F36V}. Short exposure (underneath) was used for quantification of LAP*-FKBP12^{F36V} (arbitrary units) after normalization to Poncaeu S-stained lanes (total protein loading/blotting controls; total protein loaded: 25 µg B cells, lane 1, and 100 µg for all other lanes 2-10).

D. Morphology of CEBP-dKO-LAP*-FKBP12^{F36V}-GMPBT cells with or without dTAG-13 treatment. Phase contrast (cell culture samples at day 5 post-treatment, top row) and May-Grunwald Giemsa staining (cytospins of samples at day 1 post-treatment, bottom row) images are shown. Scale bar: 50 µm. Note larger cell size and macrophage appearance after dTAG-13 treatment of GMPBT cells.

Supplemental Figure 1

Supplementary Figure S1

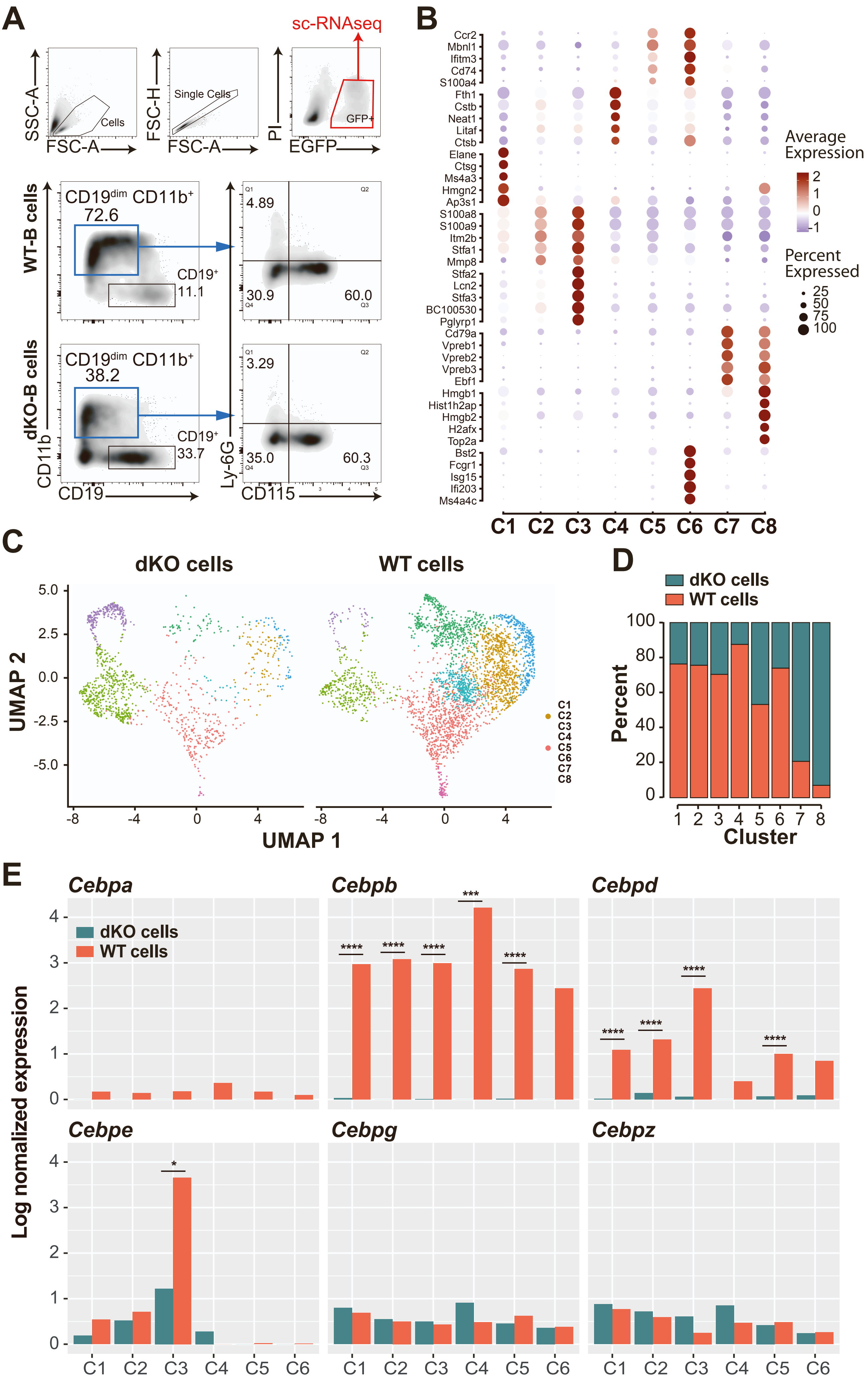

Supplemental Figure 2

Supplementary Figure S2

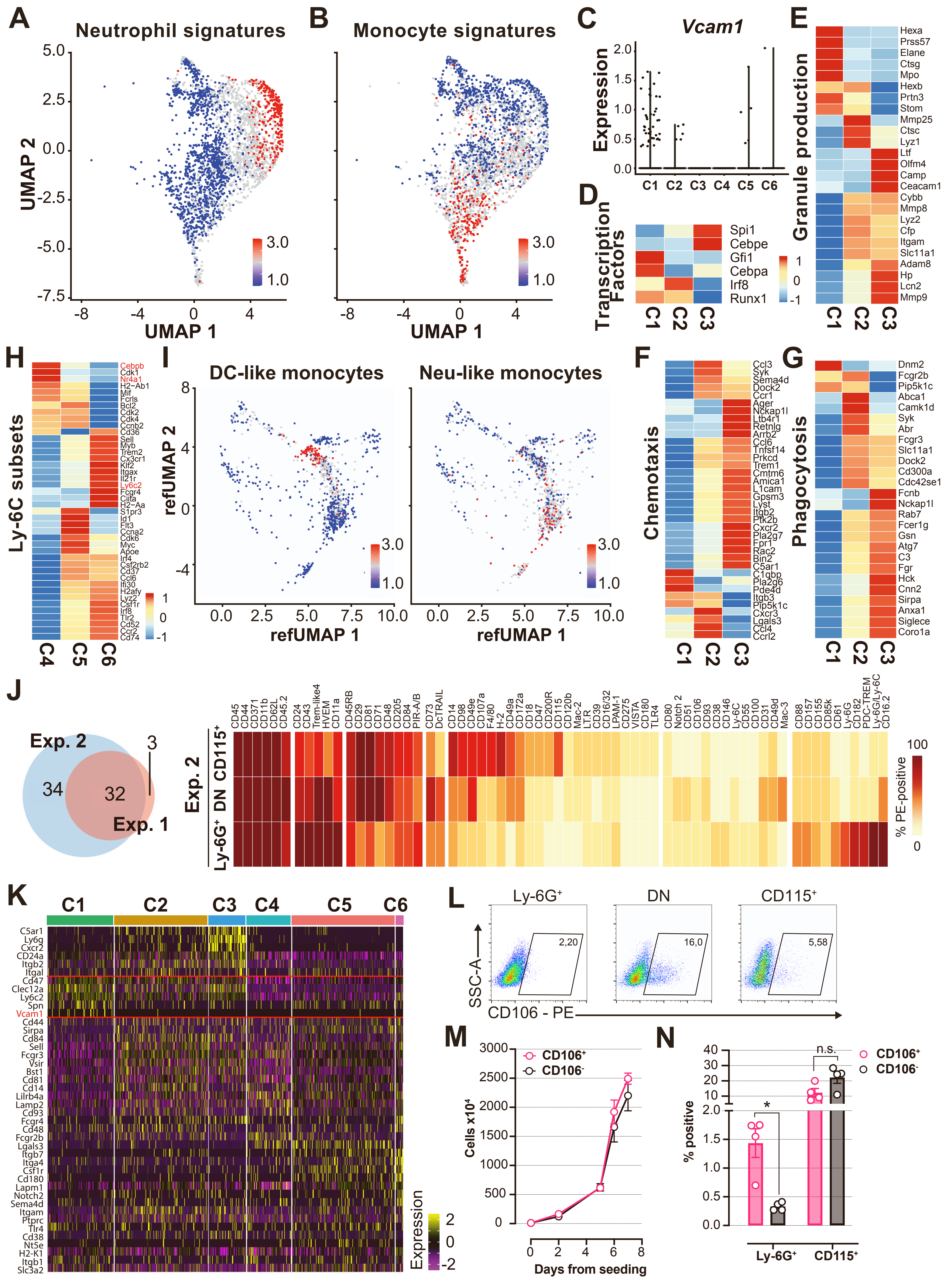

Supplemental Figure 3

Supplementary Figure S3

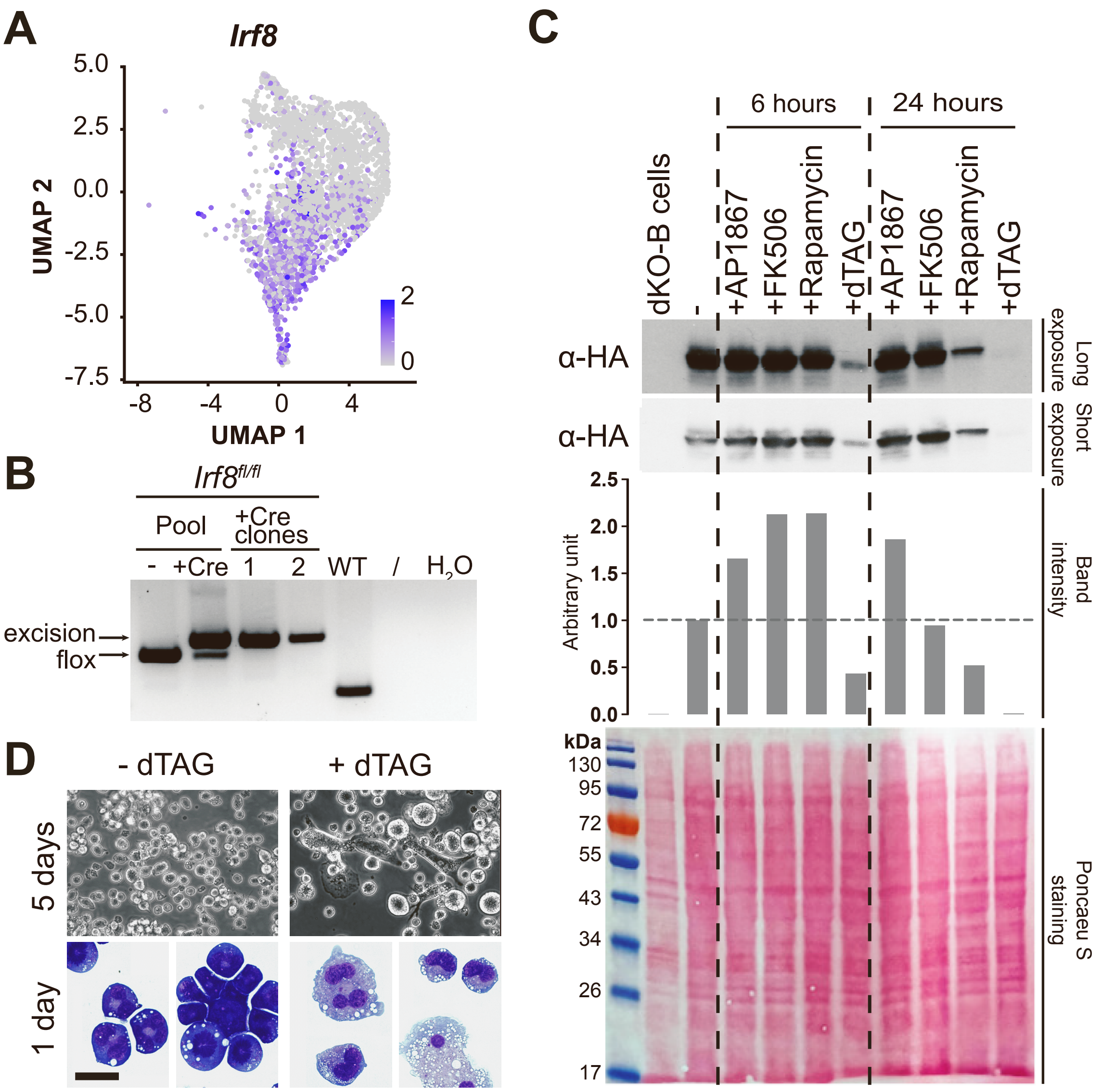

Table S1. (related to Figure 1D) Differentially expressed genes of myeloid clusters C1-C6. Genes with a corrected p-value <0.05 and FC >2 are shown.

Table S2. (related to Figure 1E) Enriched GO terms of biological processes in clusters C1-C6. Clusters marker genes with adjusted p-value <0.05 and FC >1.5 were used for this analysis.

SUPPLEMENTAL EXPERIMENTAL PROCEDURES

Vector constructs

The retroviral pMSCV-IRES-EGFP (MIEG) vector was used for ectopic C/EBPβ-LAP* expression as described previously (Cirovic et al., 2017). The C/EBPβ-LAP*-FKBP12^{F36V}-HA fusion expression construct was based on the rat C/EBPβ sequence (Gene ID #24253) and the FKBP12^{F36V}-HA was purchased from Addgene (#104371). The rat C/EBPβ-LAP* sequence was generated by PCR primers 5′-ccgctcgaggccaccatggcccaccgcctgctggcc and 3′-ctggcctcggcgggtcactgcggatccgcg from construct pMSCV-IRES-BFP C/EBPβ-LAP* (Cirovic *et al.*, 2017). FKBP12^{F36V} from pAW63.YY1.FKBP.knock-in.BFP was generated with the primers 5′-cgcggatccagcggtggaagtggtggcggagtgcaggtggaaacc and 3′-gatgtcccggactatgcataagaattccgg. Conditional STAT5A constructs were obtained from Richard Moriggl (University of Veterinary Medicine, Vienna, Austria) and were described previously (Grebien et al., 2008). Briefly, the STAT5 chimera was fused to the hormone-binding domain of the estrogen receptor (ER) variant to allow conditional activation by 4-hydroxytamoxifen (4-OH-T, Sigma-Aldrich). The GMPBT STAT5 cell lines were designated pMSCV-STAT5 WT, pMSCV-STAT5Δ749, or pMSCV-cS5.

Cells and differentiation

Cells with deletion of endogenous floxed *Cebpa* and *Cebpb* with TAT-Cre recombinase generated *Cebpa*$^{-/-}$*Cebpb*$^{-/-}$ cells (dKO-B cells). The B cells were cultured with 50 μM β-mercaptoethanol. Transdifferentiated GMPBT were selected by β-mercaptoethanol depletion as indicated previously (Cirovic *et al.*, 2017); this selection was applied in experiments involving CEBP-dKO-LAP*-FKBP12^{F36V}-GMPBT cells. After 2–3 weeks, only long-term proliferating transdifferentiated cells survived. *Irf8* knockout (KO) clones were generated from *Irf8*$^{fl/fl}$ v-Abl transformed B cells by incubation with TAT-Cre recombinase as described earlier. The Irf8 KO cell transdifferentiation was the same as that of the WT-B cells. Imatinib (Thermo Fischer Scientific; final concentration 0.4–0.6 μM), ruxolitinib (1 μM), and dTAG-13, FK506 (all, Tocris Bioscience), or AP1867 (ChemScene) (all, 1–5 μM) were added as indicated. CSF1, CSF2, and CSF3 were from PeproTech and added as indicated.

Antibody array

A pool of 10^8 GMPBT was harvested at day 32 p.i. (Experiment 1) or day 26 p.i. (Experiment 2). The harvested cells were washed and Fc-blocked using anti-mouse CD16/32 (TruStain FcX, BioLegend), then stained with a cocktail of fluorophore-conjugated anti-mouse antibodies against CD115 (AFS98, eBiosciences), Ly6G (1A8, BioLegend), and/or CD11b (M1/70, BD Pharmingen) for 20 minutes at 4°C. Lyophilized antibody plates containing specific phycoerythrin (PE)-conjugated antibodies were reconstituted following the manufacturer's instructions (LEGENDScreen™, BioLegend). The stained cells were distributed to individual wells in LEGENDScreen™ plates, washed and fixed according to the manufacturer's instructions before undergoing flow cytometric analysis using a MACSQuant analyzer (Miltenyi).

Two independent experiments were performed, with optimizations in Experiment 2, which included cell number reduction and CD11b staining exclusion (as 100% of the cells were CD11b$^+$). The measured data were processed with FlowJo following **Figure S1A** gating strategy**.** In each subset (CD115$^+$, Ly6G$^+$, Ly6G$^-$CD115$^-$ double-negative [DN]), the PE signal was overlaid against the PE signal of the equivalent isotype control to identify PE-positive percentages, which were used to compose surface antigen expression heatmaps. Negative or low-expression markers (sum PE positivity in all three subsets < 8% or <15% applied to Experiment 1 and 2, respectively) were excluded. As both experiments had similar marker expression patterns, we chose Experiment 2 for subsequent analysis.

Cytospin and Giemsa-May-Grünwald staining

Cells (1×10^4) were spun onto glass slides at 500 rpm for 5 minutes using a cytocentrifuge (Aerospray slide stainer, Wescor). The slides were air-dried, fixed with methanol for 5 minutes, immersed in May-Grünwald (Merck) solution for 5 minutes, washed with water, then immersed in Wright-Giemsa (Merck) solution for 45 minutes. After extensive rinsing in water, the slides were dried and cover-slipped (Roti-Histokitt II).

Bioinformatic analysis

Sequencing reads were demultiplexed and aligned with the mm10 genome using Cell Ranger 2.1.0 before further analyses using R 4.1.2. For the overview of the C/EBP family expression (Supplementary Figure S1E), an updated version of mm10 and CellRanger version 7.1 were used. The data was

preprocessed by the Cell Ranger pipeline 2.1.0 and analyzed using Seurat 4 (Satija et al., 2015). Raw data was filtered by removing cells with <400 features and >4000 features. Additionally, cells containing a mitochondrial read percentage of >7 were removed as potentially dead cells. The WT-B and dKO-B samples were normalized using the LogNormalize option, integrated, and scaled. Uniform manifold approximation and projection (UMAP) dimension reduction (McInnes, Healy and Melville, 2018) and clustering was performed by a shared nearest neighbor (SNN) modularity optimization-based clustering algorithm with the resolution parameter set to 0.5.

SingleR was used for automated identification and annotation of the resulting cell clusters (Aran et al., 2019). Based on the Immunological Genome Project (ImmGen) database, the clusters were assigned to cell types by comparing the expression of the cells of each cluster to the expression data deposited in ImmGen. For each cluster, marker genes were determined using MAST (Finak et al., 2015) with a corrected p-value of 0.05 and a fold change (FC) of 2. Only genes that were expressed in ≥20% in at least one group were considered.

To determine the specific cluster functions, gProfiler (Reimand et al., 2016) was applied to the marker genes of each cluster as indicated previously, except for the use of a 1.5 FC. Gene Ontology biological pathways (GO:BP) and Kyoto Encyclopedia of Genes and Genomes (KEGG) terms were used as a source and considered relevant if the corrected p-value (false discovery rate [FDR]) was <0.001.

Gene set variation analysis (GSVA) was used for targeted identification of specific functional and literature-known gene sets, i.e., their specific expression in subgroups of our cells (Hanzelmann, Castelo and Guinney, 2013), to several gene sets. Gene sets for Figure S2A,B are derived from Giladi et al. (Giladi et al., 2018) and gene sets for Figure S2I are derived from Weinreb et al. (Weinreb et al., 2020).

Developmental trajectories were inferred with Slingshot (Street et al., 2018) with cluster 1 as the starting point, as SingleR determined that it was the most precedent cluster.

To predict TFs involved in the onset of the granulocytic and monocytic developmental branch, we first identified the differentially expressed genes between clusters 1, 2 and 3 and the clusters 4, 5 and 6 (adjusted $p < 0.05$, $|FC| > 1.2$). We then applied LISA (Qin et al., 2020) to the set of up and down regulated genes in a comparative mode.

References

Aran, D., Looney, A.P., Liu, L., Wu, E., Fong, V., Hsu, A., Chak, S., Naikawadi, R.P., Wolters, P.J., Abate, A.R., Butte, A.J., and Bhattacharya, M. (2019). Reference-based analysis of lung single-cell sequencing reveals a transitional profibrotic macrophage. Nat Immunol *20*, 163-172. 10.1038/s41590-018-0276-y.

Cirovic, B., Schonheit, J., Kowenz-Leutz, E., Ivanovska, J., Klement, C., Pronina, N., Begay, V., and Leutz, A. (2017). C/EBP-Induced Transdifferentiation Reveals Granulocyte-Macrophage Precursor-like Plasticity of B Cells. Stem Cell Reports *8*, 346-359. 10.1016/j.stemcr.2016.12.015.

Finak, G., McDavid, A., Yajima, M., Deng, J., Gersuk, V., Shalek, A.K., Slichter, C.K., Miller, H.W., McElrath, M.J., Prlic, M., Linsley, P.S., and Gottardo, R. (2015). MAST: a flexible statistical framework for assessing transcriptional changes and characterizing heterogeneity in single-cell RNA sequencing data. Genome Biol *16*, 278. 10.1186/s13059-015-0844-5.

Giladi, A., Paul, F., Herzog, Y., Lubling, Y., Weiner, A., Yofe, I., Jaitin, D., Cabezas-Wallscheid, N., Dress, R., Ginhoux, F., et al. (2018). Single-cell characterization of haematopoietic progenitors and their trajectories in homeostasis and perturbed haematopoiesis. Nat Cell Biol *20*, 836-846. 10.1038/s41556-018-0121-4.

Grebien, F., Kerenyi, M.A., Kovacic, B., Kolbe, T., Becker, V., Dolznig, H., Pfeffer, K., Klingmuller, U., Muller, M., Beug, H., Mullner, E.W., and Moriggl, R. (2008). Stat5 activation enables erythropoiesis in the absence of EpoR and Jak2. Blood *111*, 4511-4522. 10.1182/blood-2007-07-102848.

Hanzelmann, S., Castelo, R., and Guinney, J. (2013). GSVA: gene set variation analysis for microarray and RNA-seq data. BMC Bioinformatics *14*, 7. 10.1186/1471-2105-14-7.

McInnes, L., Healy, J., and Melville, J. (2018). UMAP: Uniform Manifold Approximation and Projection for Dimension Reduction. 10.48550/arxiv.1802.03426.

Qin, Q., Fan, J., Zheng, R., Wan, C., Mei, S., Wu, Q., Sun, H., Brown, M., Zhang, J., Meyer, C.A., and Liu, X.S. (2020). Lisa: inferring transcriptional regulators through integrative modeling of public chromatin accessibility and ChIP-seq data. Genome Biol *21*, 32. 10.1186/s13059-020-1934-6.

Reimand, J., Arak, T., Adler, P., Kolberg, L., Reisberg, S., Peterson, H., and Vilo, J. (2016). g:Profiler-a web server for functional interpretation of gene lists (2016 update). Nucleic Acids Res *44*, W83-89. 10.1093/nar/gkw199.

Satija, R., Farrell, J.A., Gennert, D., Schier, A.F., and Regev, A. (2015). Spatial reconstruction of single-cell gene expression data. Nat Biotechnol *33*, 495-502. 10.1038/nbt.3192.

Street, K., Risso, D., Fletcher, R.B., Das, D., Ngai, J., Yosef, N., Purdom, E., and Dudoit, S. (2018). Slingshot: cell lineage and pseudotime inference for single-cell transcriptomics. BMC Genomics *19*, 477. 10.1186/s12864-018-4772-0.

Weinreb, C., Rodriguez-Fraticelli, A., Camargo, F.D., and Klein, A.M. (2020). Lineage tracing on transcriptional landscapes links state to fate during differentiation. Science *367*. 10.1126/science.aaw3381.
